# Discovery of food identity markers by metabolomics and machine learning technology

**DOI:** 10.1038/s41598-019-46113-y

**Published:** 2019-07-04

**Authors:** Alexander Erban, Ines Fehrle, Federico Martinez-Seidel, Federico Brigante, Agustín Lucini Más, Veronica Baroni, Daniel Wunderlin, Joachim Kopka

**Affiliations:** 1 Max-Planck-Institute of Molecular Plant Physiology, Department of Molecular Physiology: Applied Metabolome Analysis, Am Mühlenberg 1, D-14476 Potsdam-Golm, Germany; 2 Universidad Nacional de Córdoba, Facultad de Ciencias Químicas, Dpto. Química Orgánica, Córdoba, Argentina; 3 CONICET, ICYTAC (Instituto de Ciencia y Tecnologia de Alimentos Córdoba), Córdoba, Argentina

**Keywords:** Metabolomics, Systems analysis

## Abstract

Verification of food authenticity establishes consumer trust in food ingredients and components of processed food. Next to genetic or protein markers, chemicals are unique identifiers of food components. Non-targeted metabolomics is ideally suited to screen food markers when coupled to efficient data analysis. This study explored feasibility of random forest (RF) machine learning, specifically its inherent feature extraction for non-targeted metabolic marker discovery. The distinction of chia, linseed, and sesame that have gained attention as “superfoods” served as test case. Chemical fractions of non-processed seeds and of wheat cookies with seed ingredients were profiled. RF technology classified original seeds unambiguously but appeared overdesigned for material with unique secondary metabolites, like sesamol or rosmarinic acid in the Lamiaceae, chia. Most unique metabolites were diluted or lost during cookie production but RF technology classified the presence of the seed ingredients in cookies with 6.7% overall error and revealed food processing markers, like 4-hydroxybenzaldehyde for chia and succinic acid monomethylester for linseed additions. RF based feature extraction was adequate for difficult classifications but marker selection should not be without human supervision. Combination with alternative data analysis technologies is advised and further testing of a wide range of seeds and food processing methods.

## Introduction

Food authenticity and nutritional quality are of great interest to the food industry, producers, distributors, and consumer trust in nutritional value, origin, and production processes^1,2^. A food product can be sold at a premium price if label claims and declarations of origin and ingredient identity are certified by producers and can independently be verified by regulatory authorities or consumer´s organizations using validated analytical technologies^3,4^.

Authenticity of foods that are based on animal tissues can be traditionally verified by immunological methods. The high diversity of plant derived foods or food additives can be monitored in addition by molecular markers that may be either metabolic^5^ or genetic^6–8^. Genetic markers can be designed to authenticate species, genus, or even plant variety. Indeed, the detection of DNA is considered one of the most potent tools in food integrity research not least due to the considerable chemical stability of DNA. The use of DNA markers as diagnostic tools of validating ingredient authenticity in foods has been investigated by an increasing number of studies^6–8^.

Complementary to genetic and protein markers, metabolomics enables marker searches and validation by spectroscopic and hyphenated analytical techniques^5^, such as liquid chromatography (LC) or gas chromatography (GC) coupled to mass spectrometry (MS). Specialized software is used for compound targeted and non-targeted analyses of the large analytical data sets that are typically created by non-targeted metabolomic technologies^9^. Tools are in place to predict class membership of plant samples by statistical models that are based on metabolome profiles^10–12^. Metabolomics profiling identifies even plant varieties and crop cultivars according to their chemical composition, e.g. wheat^13^, broccoli^14^, potato^15^ or apple^16^. Moreover, metabolomics may directly assess the nutritional composition of foods. If combined with hydrolysis procedures, the amino acid composition, fatty acid content and carbohydrate composition of proteins, fats and polysaccharides can be determined. This information is indispensable to both consumers and food producers that take interest in potential health benefits and basic nutritional value of foods seen as “nutraceuticals”^17^. The large diversity of primary and specialized secondary metabolites makes plant-based food and food additives highly amenable to the search for metabolic markers of food authenticity or nutritional quality. Consequently, metabolomics approaches and especially non-targeted fingerprinting are expected to become potent tools of food authentication and discovery of food adulteration^18,19^.

**Figure 1:**
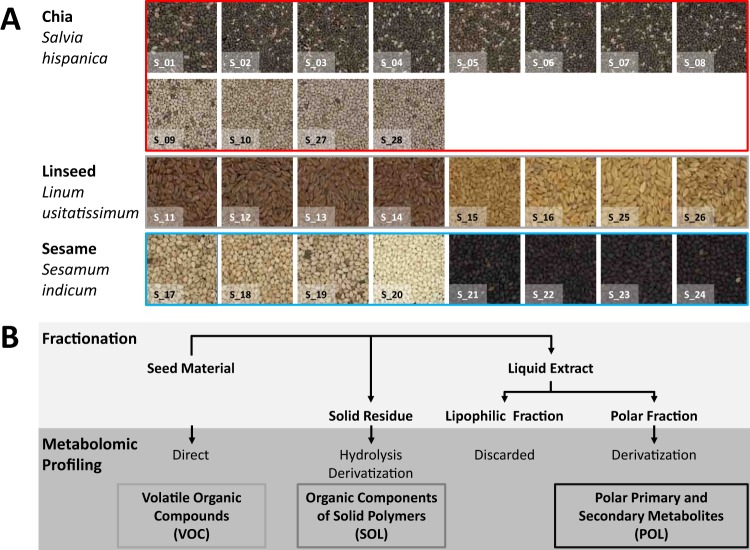
Reference samples and chemical profiling scheme for the discovery of differential metabolic markers of chia, linseed and sesame seeds. (**A**) Reference seed batches (S_01 - S_28) that were marketed for human consumption were collected from local grocery stores (Berlin, Germany) with anonymized vendor information (Table S1). Note that two colour variants of each seed type were included. All seed batches except S_20 contained the seed coat. The colour code, chia (red), linseed (grey) and sesame (blue) is used throughout the study. (**B**) Chemical fractionation and chemical profiling scheme of reference seed material. Rapid direct profiling of volatile organic compounds (VOC) was performed by headspace solid phase micro-extraction gas chromatography – mass spectrometry (SPME-GC-MS). A solid fraction (SOL) was obtained after exhaustive extraction of soluble metabolites. Solids were hydrolyzed and components analyzed by chemical derivatization and GC-MS. A polar liquid extract (POL) that was enriched for primary and small specialized metabolites was analyzed by chemical derivatization and routine GC-MS profiling. Note that the lipophilic liquid extract was omitted, because seed processing for human consumption frequently involves seed defatting and/or addition of fats from other sources.

As a test case of our current study, we analysed three types of non-processed seed materials, namely chia, linseed and sesame (Fig. 1A), which are typically added as ingredients to baked cookies or other marketed bakery products, such as crackers, breadsticks or bread. Linseed and especially chia gained consumer attention and are frequently labelled “superfoods”^20^, because these seeds may have health benefits, however, with so far limited evidence^21^. Chia (*Salvia hispanica* L.) is an annual herbaceous species of the Lamiaceae family and was traditionally cultivated in pre-Colombian Central America. After a long period of oblivion, chia has recently gained new interest mainly considering the nutritional value of their seeds. Chia seeds are rich in polyunsaturated fatty acids, vitamins, minerals, fibre and antioxidants, such as myricetin, quercetin, kaempferol, chlorogenic and caffeic acids^22–28^. Chia seeds as novel food ingredient have been approved by the European Parliament and the European Council (2009/827/EC; 2014/890/EC)^29,30^. Chia is used in bakery products, either as whole seeds or by partial substitution of wheat flour by chia flour^31,32^. Linseed (flax, *Linum usitatissimum* L.) and sesame (*Sesamum indicum* L.) are more traditional, highly nutritive seeds^33–35^. These seeds, like chia seeds, are used in bread and bakery products to improve both nutritional characteristics and consumer´s acceptance. Marketing as “superfoods” and resulting consumer interest turned chia seeds into a high value food ingredient that may become a target of fraud and adulteration. Knowledge of differentiating seed markers will benefit both manufacturers and consumers by providing means of authenticating the presence of high value seed ingredients in non-processed and processed food.

Using this test case, we established a standardized procedure for the discovery and validation of metabolic markers (in the following “markers”) that indicate food integrity, i.e. the authenticated presence of a food ingredient in raw or processed food for human consumption. For this purpose we choose an iterative process that comprises six steps. The steps included (step 1) reference material selection, (step 2) chemical fractionation, (step 3) non-targeted metabolomics analysis (step 4) non-targeted metabolomics data processing, (step 5) machine learning paired with statistical analysis for marker discovery, (step 6) chemical compound annotation. We started this process by analysis of markers that were present in non-processed chia, linseed, and sesame seeds and iterated marker discovery using wheat cookies with or without seed additions as an example of a processed food product that was in our case experimentally defined. This generalized process can be extended in step 1 to include additional food ingredients or in steps 2–4 by adding on alternative metabolomics fractionation methods and metabolomics profiling and data processing technologies choosing, for example, from the wealth of recently summarized and described methods^5^.

The analytical methods of metabolomics profiling (step 3) that we choose were previously established non-targeted profiling procedures that covered a broad range of the seed metabolome (Fig. 1B) ranging from the volatile organic fraction (VOC) of emitted fragrances^36,37^ to the polar soluble (POL) or soluble lipophilic fractions^38–41^. We included analysis of hydrolysed components of the solid food fraction (SOL) that was obtained after exhaustive extraction of soluble polar and lipid fractions^42^. In terms of analytical methods we focused in this study, for reasons of cost- and time-efficiency, on the well-established metabolomics tool box of routine GC-MS based profiling.

For data pre-processing (step 4) we selected a standardized and previously described procedure for GC-MS chromatogram data analysis that is enabled by the TagFinder software developed by our laboratory^43,44^. This non-targeted metabolomics procedure generates numerical matrices of mass feature abundances that are normalized by sample weight and abundance of an internal standard. These mass features are in statistical terms the variables that characterize the profiled seed and food samples.

We focussed specifically on step 5 and aimed to explore approaches of metabolic marker search from non-targeted metabolomics profiles. Markers among the mass features were extracted by a variant of machine learning technologies, specifically by the feature extraction process that is part of random forest (RF) analysis^45,46^. RF analysis is based on the earlier development of decision trees^46^. We choose this version of machine learning technology because other than most machine learning algorithms RFs and specifically decision trees provide human interpretable classification rules. RF analyses also allow for internally validated variable selection^45^. We characterize obtained RF based markers by subsequent statistical analysis, discuss the potential and caveats of using RF based feature extraction for metabolomics marker search and for simple classification cases where machine learning may be considered an overdesigned application we implemented a rule-based “Min/Max ratio” approach that extracts unique seed markers or markers that are highly enriched in a single seed type.

## Results and Discussion

### Volatile, polar and solid metabolome fractions differentiate non-processed chia, linseeds and sesame seeds

We first choose reference materials (step 1) with care, because this choice obviously limited the application range and quality of resulting seed identity markers. We acquired authenticated reference material of non-processed chia, linseed, and sesame seeds (Fig. 1A) from multiple independent sources that were subsequently anonymized (Table S1). This seed material was marketed and authenticated for human consumption by 15 different vendors within the European Union (EU). We analysed 28 seed batches that comprised 8 independently marketed seed batches of brown chia seeds, and 4 independent batches, each, of predominantly off-white chia seeds^24^, golden linseeds, brown linseeds, white sesame seeds, and black sesame seeds (Fig. 1A). All seed batches were free of contaminations according to visual inspection (Fig. 1A) and, except a single sesame sample (S-20), contained seed coats to account for the fact that processed food material often contains complete seeds. None of these seed batches was defatted or reported to be otherwise processed. Complete information given by the respective vendors´ packaging was retrieved and listed (Table S1). We took care to include marketed colour variants of all seed materials and, thereby, included the metabolic variation that is associated with the most frequent seed colour variants of our seed material.

We choose a non-targeted metabolome analysis approach to marker discovery (step 2). For the purpose of least biased and broad metabolome coverage we established a chemical fractionation scheme (Fig. 1B) that facilitated metabolic profiling of initially four major groups of compounds, each with different chemo-physical properties, namely the VOC, POL and SOL fractions as well as a soluble lipid fraction. First analyses of the soluble lipid fraction failed to demonstrate distinctive specialized metabolites at levels that were detectable next to the previously characterized, ubiquitous, and abundant fatty acid components^22–26,28^. For this reason we omitted the lipophilic fraction from the marker search of this study. We also decided to omit the known, distinctive fatty acid compositions of the seed material from our marker search, because lipids are frequently replaced during food processing by seed defatting and addition of alternative lipids. Thus fatty acid markers can potentially be obscured or, if done with intent, falsified.

Non-targeted metabolomics profiling of each of the VOL, POL and SOL fractions (step 3) was performed by routine gas chromatography- mass spectrometry (GC-MS) methods^37,41^. These methods had highly repeatable chromatographic separation and were in the case of the POL and SOL analyses additionally standardized by retention index (RI) calibration^47^. The GC-MS based metabolomics methods all operated at nominal mass resolution and generated highly comparable data matrices that can be compared between laboratories^47,48^. Non-targeted metabolomics data processing^43^ generated numerical data matrices of all observed mass features across the complete set of samples and their VOL, POL, and SOL fractions. The mass features that are obtained by GC-MS profiling technology are mass fragments and molecular radical ions with respective mass isotopologues of the naturally occurring stable isotopes of elements, such as ^13^C. Mass features of this study were processed by TagFinder software^43,44^ and have the properties of a nominal mass and an average retention time (VOL) or retention index (POL, SOL) within a common retention window across all samples. Our non-targeted metabolite profiling analyses yielded 14542, 26105, and 24080 mass features across all samples of the VOL, POL and SOL seed fractions (Fig. 1B). We combined these matrices to enable joined feature extraction by statistical data analysis and machine learning applications (Table S2). The typical high number of mass features of GC-MS profiling experiments is caused by multiple alternative mass spectral fragmentation pathways of the initial molecular ions. These fragmentation pathways are induced by the electron impact ionization technology used by many GC-MS instruments. The fragmentation pathways generate multiple redundant mass fragments that all represent the same compound. We maintained this redundancy in the basic data sets of our study (Tables S2 and S4) and also used naturally occurring stable mass isotopologues to support validation of our mass feature selection. For validation, we expected seed identity markers to be represented by multiple redundant mass features with similar variance importance. Variation of variance importance of redundant mass features was expected. Such variations occur depended on the relative abundance of mass features, especially if a subset of redundant mass features is close to detection limits, and depend on mass feature specificity in the presence of co-migrating compounds.

**Figure 2:**
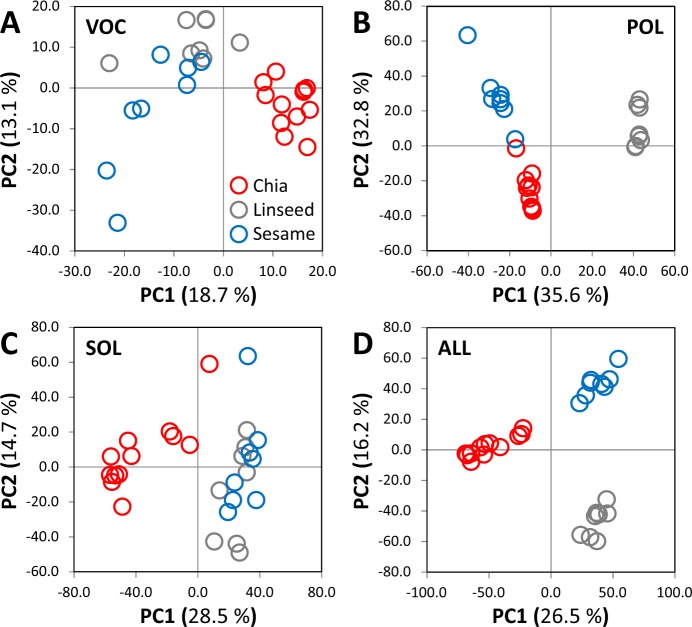
Principal component analyses (PCA) of non-targeted metabolite profiles of three chemical fractions from chia (n = 12), linseed (n = 8) and sesame seeds (n = 8). Matrices of averaged mass features across technical replicates (t) from the VOC (t = 6), POL (t = 5) and SOL (t = 2–5) profiles of the 28 seed batches (S_01 - S_28) were submitted to PCA analysis (Table S2). The first two components of each separate PCA, VOC (**A**), POL (**B**), SOL (**C**), and a PCA of the combined data sets (**D**) are plotted. Log_10_-transformed mean-centred ratios of each mass feature were calculated prior to PCA. Missing value (NA) replacement was an estimate of the detection limit (Fig. 4) before calculation of log_10_-transformed ratios.

Separate principal component analyses (PCA) served as a first overview of the metabolome analyses and demonstrated that all fractions contained seed specific information among the major variances of the data subsets (Fig. 2). The POL fraction appeared to be most informative for the differentiation of non-processed seed material (Fig. 2B). All fractions carried valuable seed-discriminatory information as is exemplified by enhanced seed type separation by PCA of a combination of all data (Fig. 2D) compared to the PCA of the POL profiles alone (Fig. 2B). The first two principal components of the PCA of the POL profiles covered the largest percentage of total variance, namely 68.4%. In comparison, the first two principal components of the SOL and VOL fractions contained only 43.2% or 31.8% of total variance, respectively. In the following, we used information of all fractions for marker search by statistical analyses and RF based feature selection procedures.

### RF based feature selection enables discovery of markers of non-processed seeds

**Figure 3:**
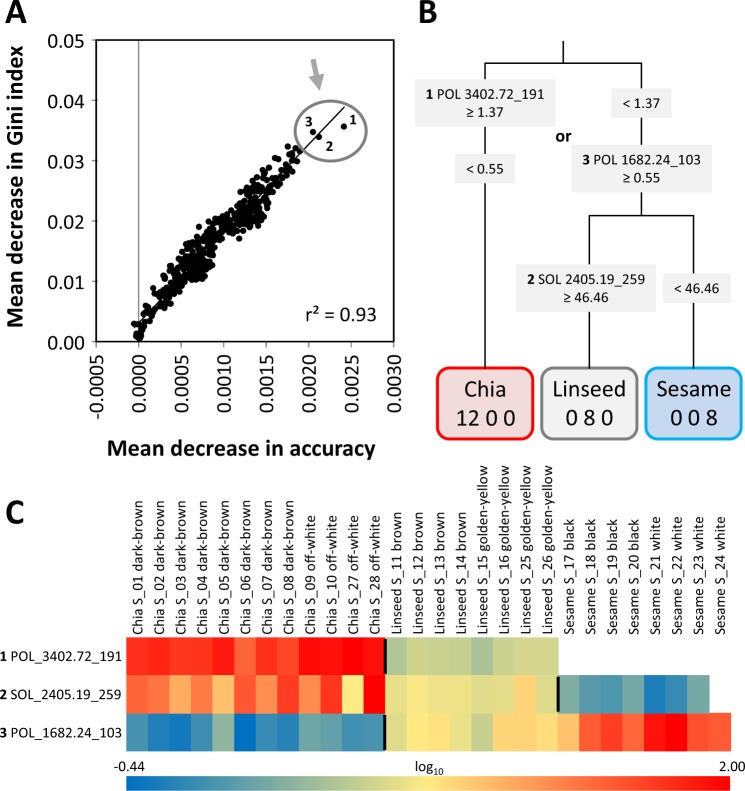
RF based analysis of seed identity markers from mass features of non-targeted metabolite profiles of three chemical fractions from chia, linseed and sesame seeds. Analysis of variable importance using the mean decrease of Gini index and mean decrease of accuracy measures of random forest (RF) analyses. The three most important variables, i.e. mass features, are indicated by circle and arrow. Ten random forest analyses were performed by repeated random selection of 14 training profiles from 28 seed batches. The mean decrease in Gini index and mean decrease in accuracy measures of mass features were averaged across the random forest analyses. None of the classification models had errors. Linear correlation, r² of a Pearson’s correlation coefficient, of the averaged variable importance measures is inserted. Averaged normalized abundances of the mass features across technical replicates of the VOC, POL and SOL analyses were used (Table S2). Mass features were pre-selected according to 1-way ANOVA (P < 10^−5^) significance for the distinction of seed types. Redundancy of mass features was reduced by selecting the most significant feature with least missing values among multiple mass features of the same compound. (**A**) Decision tree representation of two rule sets that distinguish seed material without false classifications. The rules were based on the three most important mass features. Mass features are reported by analysed fraction, chromatographic retention time RT (VOC) or retention index RI (POL, SOL) and nominal mass. The numerical values in the tree are the thresholds of normalized abundances for partitioning of the seed samples. (**B**) Heat map representation of the normalized abundances of the three most important mass features. The normalized abundances were maximum scaled and log_10_-transformed for visualization, maximum (red), mean (yellow), minimum (blue), and non-detected (white). Vertical bars within the heat map indicate the partitioning depicted in panel (B).

In view of the in part largely overlapping clusters of chia, linseed, and sesame seed samples that were apparent in PCA analyses, e.g. (Fig. 2A,C), we did not perform already well described approaches of feature selection for marker identification, such as orthogonal partial least squares projections to latent structures (O2PLS) or loadings analysis of the first principal components. To add options to the current tool box of metabolic marker search^49^, we selected mass features by RF based machine learning (Fig. 3). For this process we filtered our complete data set by 1-way ANOVA to enrich for mass features with highly significant information (*P* < 10^−5^) in regard to the distinction of seed classes either excluding or including the colour variants of each seed type (Table S2; columns AV-AW). We also largely eliminated redundancy of mass features by selecting only the most significant feature with least missing values among the multiple redundant mass features of each metabolite. We trained RF classification models by repeated random selections of half of the data set. All repeatedly trained models classified non-processed chia, linseed, and sesame seed material without error. Ranking of variable importance according to the mean decrease in accuracy or Gini index measures of our trained classification models varied with the repeated random choices of training data subsets. For this reason, we repeated our RF models and averaged the mean decrease in accuracy and Gini index scores until their ranking and the linear correlation between the two variable importance measures stabilized (Fig. 3A). This process yielded a large number of important variables (Fig. 3A). We selected the three most important mass features for manual inspection, as exemplified by a heat map display (Fig. 3C). We concluded that already the three most important mass features enabled multiple alternative unambiguous seed classification. Mass features were named by fraction, retention index, and nominal mass, for example, POL_3402.72_191 that was later annotated to represent *trans*-rosmarinic acid of chia seeds. This mass feature alone classified all seed samples unambiguously by absence in sesame seeds, low levels of in linseeds and high levels in chia (Fig. 3C). Note that low levels of this mass feature in linseeds were caused by a co-migrating compound that also generated a fragment of m/z = 191 but was not *trans*-rosmarinic acid. In addition, rule sets based on two mass features, e.g. POL_3402.72_191 (*trans*-rosmarinic acid) and SOL_2405.19_259 (non-annotated metabolite) or POL_1682.24_103 (non-annotated metabolite) and SOL_2405.19_259, as depicted by a joined decision tree (Fig. 3B), enabled correct classifications that are validated by two rule-sets. Our data obviously contained multiple alternative annotated and non-annotated markers for the unambiguous classification of non-processed seeds. We concluded that elaborate machine learning may be applied but is likely not required in this case where single metabolites or combinations of two metabolites classified seed types unambiguously.

### Feature selection by Min/Max ratios enables ranked feature extraction of unique markers

**Figure 4:**
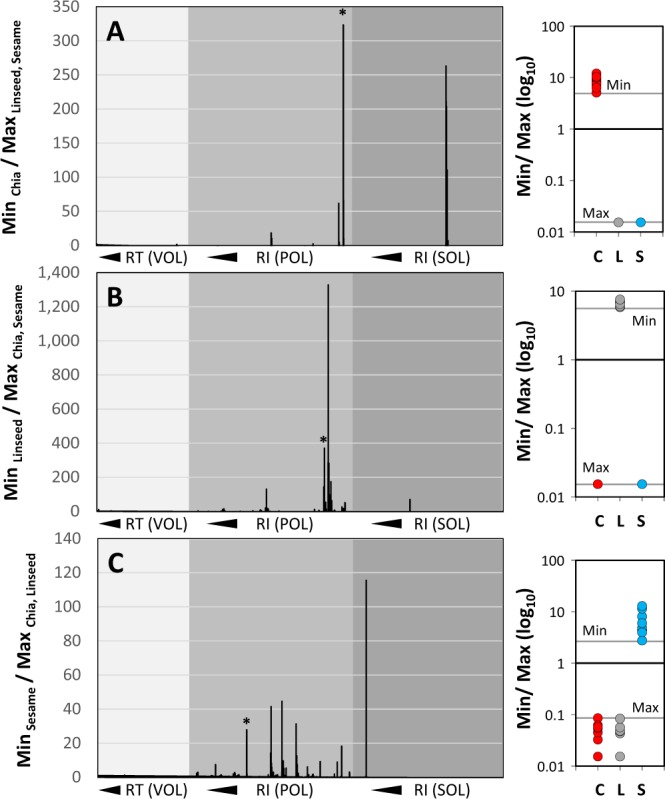
Minimum over Maximum ratios of mass feature abundance distributions from non-targeted metabolite profiles of three chemical fractions from chia, linseed and sesame seeds. Ratios of minimum (Min) abundances of mass features observed in one seed type over the maximum (Max) abundance in the other seed types are plotted left to right according to retention time (RT) of the VOC analysis (light grey underlay) or according to retention index (RI) of POL (middle grey) and SOL (dark grey) profiles, (**A**) Min_Chia_/Max_Linseed, Sesame_, (**B**) Min_Linseed_/Max_Chia, Sesame_, and (**C**) Min_Sesame_/Max_Chia, Linseed_. Inserts to the right illustrate exemplary abundance distributions using the mass features that are indicated by a star (*) to the left. Note that the plotted Min/Max ratios represent a measure of the gap between non-overlapping abundance distributions. Missing values were substituted before ratio-calculations by an estimate of the detection limit.

For cases of data sets that enable unambiguous classification by multiple markers that are in part unique or highly specific to one or more sample types, we established a simple rule-based method to select and rank the most important mass features from a multitude of options. We argued that an ideal marker of seed identity should be present at high abundance in one seed type and absent, i.e. in the case of a unique marker, or present at only very low abundance, i.e. in the case of a highly specific marker, in the other seed types. According to this rationale we selected mass features by abundance ratios. For this purpose, we calculated ratios of the minimum observed abundance (Min) of mass features across all replicates of one seed type, e.g. all measurements of brown and off-white chia seeds (Fig. 4A), relative to the maximum abundance (Max) across all remaining measurements of other seed types, i.e. all samples that are not chia seeds (Fig. 4A). Missing values were replaced by an estimate of the detection limit of our VOC, POL and SOL analyses. We defined these estimates as the minimal observable normalized abundance separately in each of the three analysed fractions. The Min/Max ratio approach enabled ranked mass feature selection according to largest relative abundances. True positive observations, e.g. chia seeds, were clearly distinguished from all true negative observations in other seed types as was visualized by examples (Fig. 4, right hand panels). We performed this calculation for each seed type using the complete data set (Table S2). The display of these ratios indicated the presence of few, however, highly abundant marker substances of each seed type, chia (Fig. 4A), linseed (Fig. 4B), and sesame (Fig. 4C).

**Figure 5:**
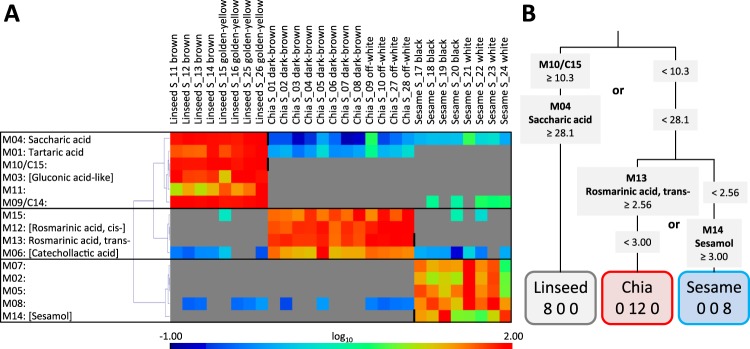
Min/Max - based selection of seed identity markers among mass features of non-targeted metabolite profiles of three chemical fractions from chia, linseed and sesame seeds. (**A**) Heat map representation of the normalized abundances of 15 Metabolites, M01-M15, that are markers of seed identity and grouped by seed type. The compounds were selected according to top 15 Min/Max ratios of mass features. Compound annotations by mass spectral match alone are reported in square brackets. Compounds without clear match were given an M identifier, e.g. M10 (also found as C15 in the subsequent analyses), and documented by mass spectrum and retention index (Supplemental Data File S5). The normalized abundances were maximum scaled and log_10_-transformed after mean centring for heat map visualization, maximum (red), mean (green), minimum (dark blue), and non-detected (gray). Note that this approach yields only “positive” markers of seed identity. Vertical black bars within the heat map indicate the sample partitioning rules of panel (**B**). Hierarchical clustering was by r² distance metric (Pearson’s correlation) and complete linkage. (**B**) Decision tree representation of two rule sets that distinguish three classes of seed material without misclassification. The rules were based on four metabolites, M04 (saccharic acid), M10/C15 (non-identified), M13 (*trans*-rosmarinic acid), and M14 (sesamol). The numerical values are the partitioning thresholds.

We selected fifteen marker substances with top Min/Max ratios larger than 10-fold across all seed types and metabolite fractions (Fig. 5, Table S3). The marker substances were typically represented by two or more mass features. The VOL fraction yielded no marker with Min/Max ratios of two or more mass features larger than 10-fold. The SOL fraction yielded two. The POL fraction proved to be the most informative (Fig. 4). We obtained four markers of chia seeds, six of linseeds and five of sesame seeds (Fig. 5A). All compounds classified non-processed seed types without error as “positive” markers of identity. As “positive” markers of seed identity, we define those compounds that are either unique or highly specific to a seed type (Fig. 5) opposed to the concept of a “negative” marker that may be present in all seed types except one. Combinations of rules that were based on two or three of these Min/Max ratio markers enabled correct unambiguous classifications that were validated by multiple rule-sets (Fig. 5B).

### Chemical compound annotation

The metabolomics annotation process of chemical compounds is still not fully automated and must be manually supervised^50^. Annotation is therefore time consuming and should be focused only to the relevant mass features from non-targeted metabolome profiling studies. In the current studie we selected of the most informative mass features and subsequently annotated only these metabolites.

For the purpose of chemical compound annotation (step 6) we manually retrieved complete mass spectra from selected chromatogram files that had highest mass feature abundance and fewest co-migrating compounds. We compiled the mass spectra and retention index information of the top-ranking markers, M01 - M015, in a file format that is ready for mass spectral comparison and analyses (Supplemental Data File S5). Based on this information, we annotated seven metabolites by mass spectrum and/or retention index match (Fig. 5), eight remained non-identified. Among the annotated metabolites, we found sesamol (M14), i.e. benzo[*d*][1,3]dioxol-5-ol, as one of the five sesame seed markers, next to the non-identified metabolites, M02, M05, M07, and M08. Chia seeds were identified by chromatographically separated *cis*- and *trans*-rosmarinic acid (M12 and M13), i.e. (*R*, *E/Z*)-3-(3,4-dihydroxyphenyl)-2-((3-(3,4-dihydroxyphenyl)acryloyl)oxy)propanoic acid, and by their biosynthetic precursor, catechollactic acid (M06), i.e. 3-(3,4-dihydroxyphenyl)-2-hydroxypropanoic acid, plus non-identified metabolite (M15). Linseeds contained characteristic amounts of tartaric acid (M01), saccharic acid (M04), a hexonic acid (M02) similar to gluconic acid and non-identified glyco-conjugates M09, M10, and M11 (Fig. 5).

### Analysis of potential processing-independent seed identity markers in experimental cookies

For our second iteration of seed identity marker search we used experimental wheat cookies that contained either 15 ± 5% (w/w) whole seeds or various amounts of defatted seed flour. Both materials are examples of typical seed additions to food material. We added increasing amounts, 5, 10, 15, or 20% (w/w), of defatted seed flour to test the potential of markers for the quantification of added seed material. In this iteration, we only profiled the POL fraction because it previously proved to be the most informative. We again created a non-targeted metabolomics data set of, in this case, 19761 mass features (Table S4).

We first checked the presence of markers of non-processed seeds, i.e. processing-independent markers, in our cookie material. We found the chia markers *trans*-rosmarinic acid (M13) and catechollactic acid (M06), however, only if high amounts of defatted chia seeds were added to the experimental cookies. Similarly, the non-identified sesame markers M05 and M07 were present only at low abundance. Two linseeds markers were sufficiently abundant even in cookie material with 5% defatted seed flour. These linseed markers were M09 and M10 and were further supported by trace amounts of tartaric acid (M01) and saccharic acid (M04). In conclusion, several processing-independent markers were present in experimental cookies, but in most cases, except M09 and M10, the compounds were too dilute or possibly heat-unstable for direct analysis of food material.

### RF based feature selection revealed potential processing-dependent seed identity markers in experimental cookies

Alternative to the search of previously identified markers, we iterated seed identity marker search (step 5) without applying previous knowledge of processing-independent markers. We again created RF models based on repeated random selection of training subsets from the full non-targeted metabolome data set and analysed variable importance by averaging mean decrease in accuracy and Gini index measures across the iterated trained models. In this case, we used the complete available data set (Table S4) and did not apply pre-filtering by 1-way ANOVA. Test RF models that included the prediction of the exact amount of 5–20% seed material had high overall error and were therefore not further pursued. Instead, we predicted only four classes, namely control cookies, and cookies with either chia, linseed or sesame seeds irrespective of the quality (defatted seed flour or whole seeds) or amount of added seeds. Eventhough we expected that wheat cookies with low amounts of seed material should be more difficult to classify than the non-processed seeds, first test models had moderate error rates of approximately 6.5%. Therefore we continued with FR based mass feature selection.

We selected the 84 most important mass features from the total set of 19761 by a take-the-top variable-out approach while monitoring the decay of the overall classification error of the iterated models (refer to method section). These 84 mass features corresponded to 14 compounds. Two of these compounds were identical with previously identified markers of non-processed linseeds, M09 and M10 (Table S4). Besides additional non-identified compounds (Supplemental Data File S5) we found the potential markers, succinic acid monomethylester, 4-hydroxybenzaldehyde, a methyl-inositol with mass spectral similarity to pinitol, a pentitol, galactinol, raffinose, a trisaccharide with mass spectral similarity to melezitose, and two fatty acids, oleic acid and linoleic acid (Table S4). Manual curation of the complete data set revealed that the current RF based variable selection procedure was not exhaustive. Additional potential marker substances were found by scoring the linear correlation coefficients of mass feature abundance to the added amount of defatted seed material (Table S4, columns DP-DU). Correlative approaches towards marker search have been previously applied^49^ and are applicable in cases, such as our cookie test case, where increasing amounts of food material are added. From these additional compounds we selected tyrosol, i.e. 4-(2-hydroxyethyl)phenol, and *myo*-inositol for subsequent analyses. This selection was based on chemical proximity to the previously identified potential markers.

**Figure 6:**
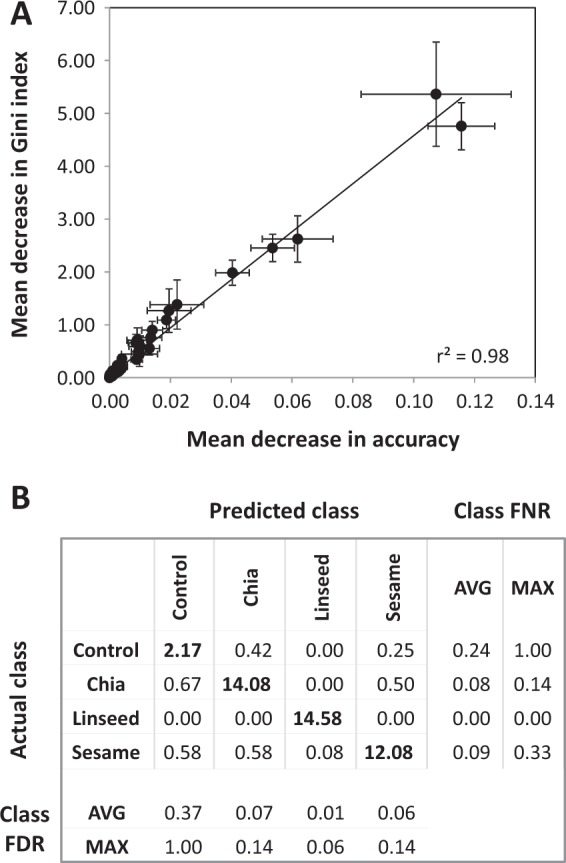
RF based selection of seed identity markers from mass features of non-targeted metabolite profiles of experimental bakery products that were prepared with or without additions of chia, linseed, or sesame seeds. (**A**) Analysis of variable importance by mean decrease of Gini index and mean decrease of accuracy measures (means ± standard deviations) of 12 random forest analyses using 84 pre-selected processing-dependent mass features and eight manually added mass features containing previously identified markers of non-processed seeds. These mass features were selected from 19761 mass features of a non-targeted metabolite profiling analysis of polar extracts from experimental cookies that were prepared with 5, 10, 15, or 20% (w/w) defatted seed flour or 10 or 20% (w/w) whole seeds (Supplemental Table S4). The classification models predicted four classes, cookies without added seeds and cookies with chia, linseeds or sesame seeds irrespective of amount of added seed material or seed pre-processing. The importance of top mass features was ranked according to mean decreases in accuracy (Supplemental Table S4). (**B**) Characterization of the trained classification models by a confusion matrix, class false negative rates (FNR) and class false discovery rates (FDR). Averages (AVG) and maxima (MAX) of class FNR and FDR were calculated from the individual confusion matrices of 12 classification models that were trained from 46 random samplings of a total set of 93 profiles of cookies without added seeds (n = 5) and cookies with chia (n = 28), linseeds (n = 30) or sesame seeds (n = 30). The overall classification error was 6.70 ± 3.27% (mean ± standard deviation).

For further analysis, we combined the 84 RF selected processing-dependent mass features, including C14/M09, C15/M10, with mass features of the previously checked but low abundant processing-independent markers, M01, M04, M05, M06, M07, and M13, and of the manually added compounds, tyrosol (C13) and *myo*-inositol (C16). The resulting set of 92 mass features of 22 compounds from the POL metabolome profiles of experimental cookies was used for RF classification of seed type additions to wheat cookies (Fig. 6, Table S4). We again generated repeated RF classification models based on random selections of training subsets and repeated training RF models until the averaged measures of variable importance, mean decrease in accuracy and mean decrease in Gini index, were largely stable and highly correlated (Fig. 6A). On average, the trained RF models had 6.70 ± 3.27 overall error % (mean ± standard deviation). This overall error of RF models based on the 92 selected mass features was similar to the error of models that used the complete data set (cf. above). Confusion matrices that were averaged across the repeated RF-models (Fig. 6B) indicated that the presence of linseeds in wheat cookies was classified best with zero false negative rates (FNR) and maximally 0.06 false discovery rates (FDR). Diagnosis of chia or sesame seeds had slightly higher but still small errors, with FNR 0.08 or FNR 0.09 and FDR 0.07 or FDR 0.06, respectively. As was expected, control cookies were the most difficult to distinguish with average FNR 0.24 and average FDR 0.37. Most of the misclassified profiles were of cookies that had no or low amounts of added seeds. The variance of the overall error %, namely ± 3.27% standard deviation, across the repeated RF-models may be explained by the varying random choice of different proportions of difficult to classify POL metabolome profiles with no or low amounts of seeds in each of the RF iterations.

### Curation and characterization of processing-dependent seed markers in experimental wheat cookies

**Figure 7:**
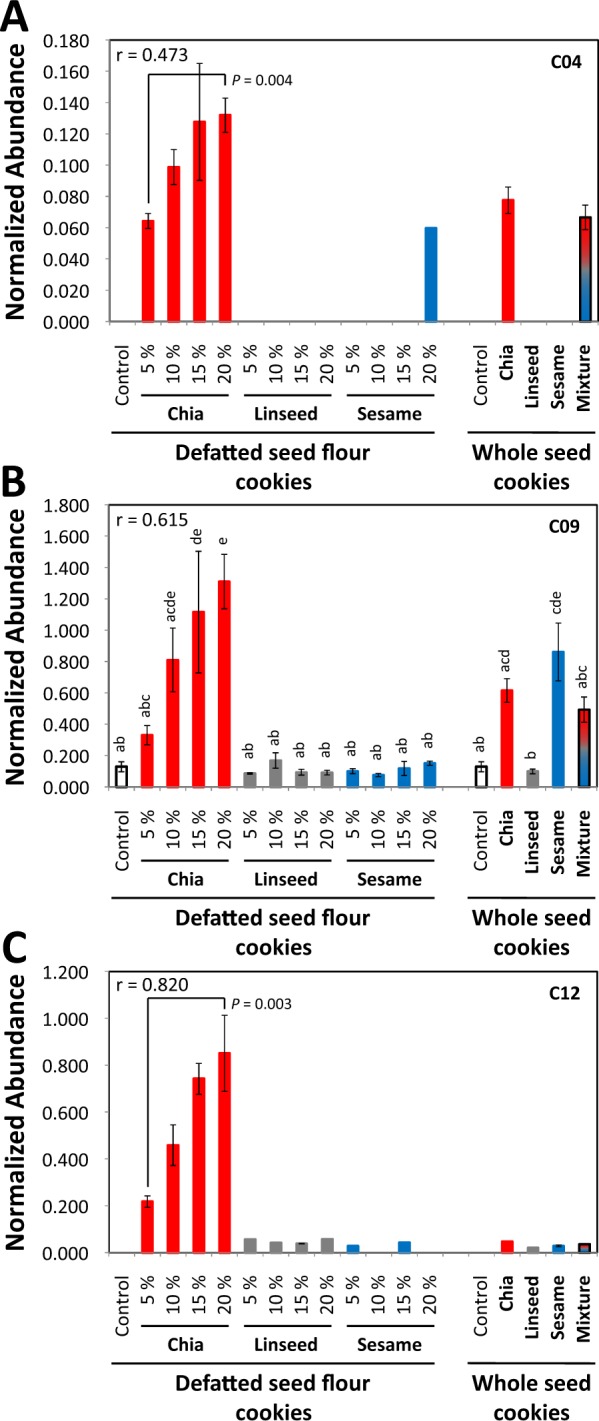
Characterization of chia seed identity markers in experimental bakery products. Non-targeted metabolite profiling analyses of polar extracts were generated from experimental cookies that were prepared, left to right, with 5, 10, 15, or 20% (w/w) defatted seed flour of single seed types, 15 ± 5% (w/w) whole seeds of single seed types, or an equal (w/w/w) mixture of whole seeds. Inserts show the Pearson’s correlation coefficients of weight percentage of the seed types and normalized abundance of marker compounds. Normalized abundances are means ± standard error (n = 4–5), bars without whiskers are single observations. (**A**) Compound C04: 4-hydroxybenzaldehyde. (**B**) Compound C09: a tri-saccharide with best match to melezitose. (**C**) Compound C12: a monomethylinositol with best mass spectral match to pinitol. Tukey’s test (lowercase letters) was performed, if applicable. If the compound was not detectable in control samples, exemplary *t*-tests are included (*P*).

**Figure 8:**
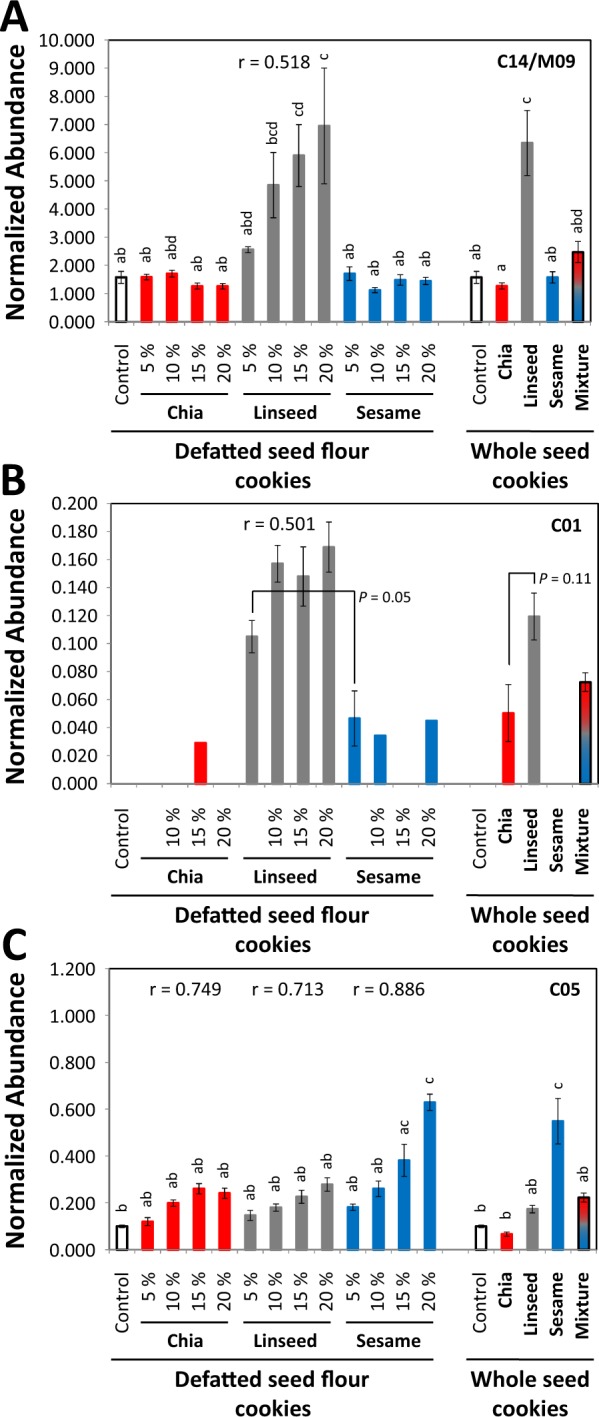
Characterization of linseed markers and a general seed identity marker in experimental bakery products. Non-targeted metabolite profiling analyses of polar extracts are depicted as described (Fig. 7). (**A**) Compound C14 also identified as non-processed seed marker M09: a non-identified marker compound. (**B**) Compound C01: monomethylsuccinate. (**C**) Compound C05: a pentitol with best mass spectral match to xylitol. Tukey´s test (lowercase letters) was performed, if applicable. If the compound was not detectable in control samples, exemplary *t*-tests are included (*P*).

**Figure 9:**
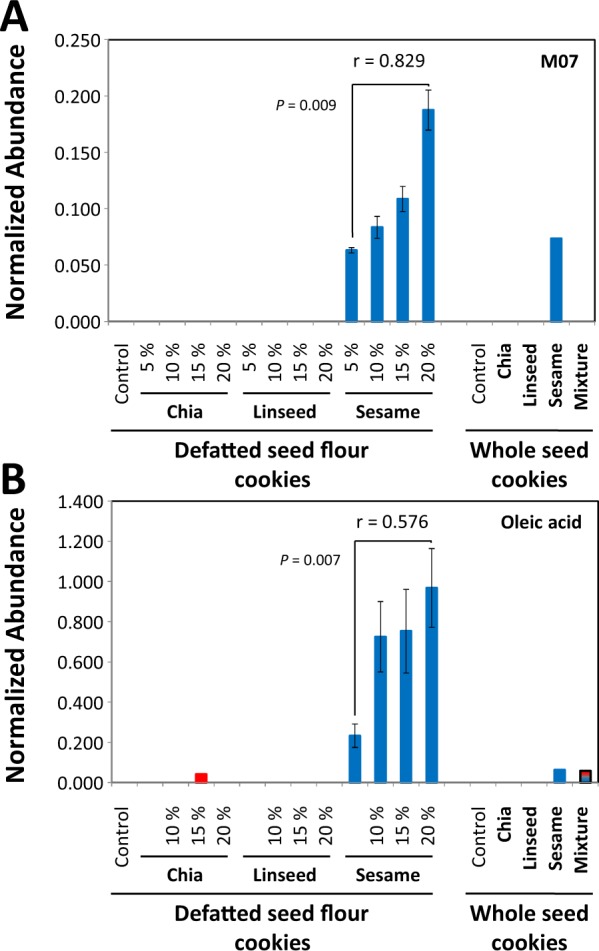
Characterization of a sesame marker and properties of oleic acid in experimental bakery products. Non-targeted metabolite profiling analyses of polar extracts are depicted as described by (Fig. 7). (**A**) Compound M07 was selected as a sesame marker by analyses of non-processed seeds (Fig. 5). M07 was detectable in experimental cookies. M07 is a non-identified compound. (**B**) Oleic acid was eliminated as a potential seed marker by manual curation because a ubiquitous fatty acid obviously has no specificity as a sesame marker. Note that non-targeted analyses may yield potential but non-specific markers, such as in this case oleic acid. Without careful curation, such a marker would lead to misclassifications of food material. Exemplary *t*-tests are included (*P*).

To evaluate the machine learning based choice of marker substances by RF classification models we manually assessed their variable importance measures and curated the selected compounds (Table S4, Figs 7–9). As was expected, the mass features of the added low abundant processing-independent markers, M01, M04, M05, M06, M07, and M13, had low variable importance for the RF classification models with ranks according to mean decrease in accuracy ≥58 of all 92 variables (Table S4, columns DI-DN). The manually added tyrosol (C13) and *myo*-inositol (C16) ranked 18^th^ and 9^th^. The high variable importance of the manually added compounds indicated context dependency of RF based marker selection. All remaining 14 compounds had at least one mass features that ranked in the top 35 of important variables. The importance of multiple mass features of single compounds was variable and likely dependent on their respective abundance. Variable importance can also be influenced by the presence of interfering mass features from co-migrating compounds. For further characterization of the discovered markers we compared the marker abundance in experimental wheat cookies with added defatted seed flours to cookies with whole seeds of a single seed type or an equal mixture of all three (Figs 7–9).

#### Markers of chia seeds

We found 4 processing-dependent markers of chia seeds, C04, C09, C12, and C13, in experimental wheat cookies, which were correlated to the amount of added seed flour. 4-Hydroxybenzaldehyde (C04) ranked top 2 according to mean decrease of accuracy of our RF models. 4-Hydroxybenzaldehyde (C04) correctly indicated the expected presence of chia seeds in all tested materials. It was, however, present in a single 20% sesame seed flour cookie (Fig. 7A). The manually selected tyrosol (C13) also correctly indicated the presence of chia seeds in all tested materials but was present at high background levels in wheat cookies. This background obscured the presence of chia in whole seed cookies and 5% chia seed flour cookies (Table S4). The trisaccharide C09 was a useful chia marker only in cookies with defatted seed flour. In whole linseed and sesame seed cookies this or similar co-migrating trisaccharides were present (Fig. 7B). The methylinositol C12 only appeared at high abundance in chia seed flour cookies (Fig. 7C).

#### Markers of linseeds

We extracted 4 processing-dependent linseed markers, succinic acid monomethylester (C01), C02, raffinose (C08), and C10, and in addition the two, already previously mentioned, non-processing dependent markers, M09/C14 and M10/C15, of linseeds from our experimental wheat cookie data set. The abundance of only three of these markers, succinic acid monomethylester (C01), raffinose (C08), and M09/C14, was correlated at r² > 0.450 to the amount of added seed flour. The remaining markers indicated only the presence but not the amount of added linseed flour. Even though non-correlated to the amount of added seed material, the mass features of non-identified compound C02 ranked first and third according to mean decrease of accuracy of our RF models. C02 and M10/C15 indicated the presence of linseeds in both, whole seed and seed flour cookies (Table S4). Likewise, non-correlated C10 ranked 13^th^ but indicated the presence of linseeds correctly in all tested materials (Table S4). M09/C14 ranked fifth, occurred at high background levels in wheat cookies but indicated the presence of linseeds correctly in all tested materials (Fig. 7A). Similarly, succinic acid monomethylester (C01) ranked fourth, occurred sporadically as a background in other material but correctly indicated the presence of linseeds in whole seed and seed flour cookies (Fig. 7B). Raffinose (C08) had high background levels in most materials but accumulated most in material that contained linseeds (Table S4).

#### Markers of sesame seeds

Feature selection by RF modelling yielded fatty acids, namely linoleic acid (C06A) and oleic acid (C06B), that acted as potential markers of the presence of sesame seed flour in wheat cookies (Table S4, Fig. 9B). The fatty acids were likely derived from a highly polar lipid fraction of sesame seeds that was co-fractionated into the POL fraction of our chemical analysis scheme. As was expected, these fatty acids were not applicable to other food material and can lead to misclassifications (Fig. 9B). Instead, previously selected non-processing dependent sesame seed marker M07 was correlated to the amount of added seed flour and correctly classified all materials (Fig. 9A). M07 was however not selected by our RF model-based feature selection process.

#### Other selected compounds

Our feature selection process also yielded potential marker compounds with unexpected properties, such as C03 (Table S4) that ranked 12^th^. Upon manual inspection C03 had no obvious specificity for a seed type or large differences of abundance between seed types that would explain the high feature importance for our RF models, except that it was absent from all control cookies. Inversely, we found high-ranking compounds that were correlated to the amount of any type of added seed material. The pentitol C05 (Fig. 8C) and *myo*-inositol (C16) were common to all seed types and, therefore, not suited to differentiate. Both compounds were present at low abundance in control cookies, correlated to the amount of added seed flour, and were most abundant when sesame seed material was added.

## Conclusions

### On the use of machine learning technology for variable importance assessment

Our study demonstrates that machine learning technology may support marker search, here the search for markers of food integrity as exemplified by the test case of three seed types that are media-hyped as superfood additions to diverse processed food materials. We used variable importance measures of RF models to select potential seed markers, similar to one of our previous applications^12^. The two available measures mean decrease in accuracy and mean decrease in Gini index were highly correlated and can be seen as largely equivalent for the scoring of variable importance. Aggregating across repeated RF training models stabilizes the correlation between the two variable importance measures and respectively derived rankings (Fig. 3, Fig. 6). However, even if the models perform without classification error (Fig. 3) we suggest not to use feature extraction by RF modelling as a stand-alone approach. We show that feature extraction by RF modelling in the implementations of our current study may not be exhaustive (Fig. 5, Table S4) with the current hyperparameter settings applied in this study. Additional markers can be found that may be obscured either by a high number of potentially important variables (Fig. 3A) or that may be overlooked due to the context of all available variables in the data set, as we exemplified by addition of manually extracted mass features to our second round of RF analysis of seed identity in wheat cookies. We attempted to account for the context dependency by choosing a take-the-top variable-out approach of feature selection from subsequently iterated RF models^12^. This approach still overlooked potential markers such as tyrosol or *myo*-inositol that we found by simple correlative manual data analysis. Additional statistical or machine learning approaches and developments designed to optimize classification models or feature selection, such as the hyperparameter tuning of RF models^51^, may enhance comprehensive extraction of all possible markers from complex data sets. Obviously, these approaches do not necessarily need follow strict cost- and time-saving principles of data mining as, for example the Min/Max ratio scoring (Fig. 4) or the rather basic feature extraction procedure by Pearson’s correlation to the amount of added seed material, which we applied to complement our current RF based approach. Nevertheless, correlation analysis demonstrated that identified markers have the potential to indicate the quantity of the added seed material or that the current potential markers may at least be useful to indicate the presence of seed types in wheat cookies (Table S4).

### On caveats

The choice of pre-filtering of data sets obviously determines the range of variables that can be selected. In our search for markers of non-processed seed types the pre-filtering by 1-way ANOVA filtering may have eliminated potential markers. Also the choice of classes for RF modelling will bias the importance of variables. For example, we choose to classify the absence or presence on three seed types in wheat cookies irrespective of the added amount and quality of seed material. Because our data set was likely too small, classification of the amount of added seed material with an acceptable error was not an option. As a consequence, resulting markers were not necessarily correlated to the amount of added seed material. While such markers are useful for the purpose of scoring the presence or absence of seed material, other markers that correlate in abundance to the amount of added seed material can be considered superior. Our feature selection approach also found markers that indicated the presence or absence of general seed material without specificity for single seed types. While inferior to seed specific markers, such general seed markers may be useful to establish a metabolic pattern of normalcy in combination with the presence or absence of seed specific makers. As a note of caution, ubiquitous compounds that do not even have specificity for general seed additions such as fatty acids should clearly be avoided (Fig. 9). Non-supervised marker selection found in our test case such ubiquitous compounds, namely oleic acid or linoleic that may only mimic specific markers and should be omitted. Clearly, stable markers that are independent of food processing technologies and that are present already in non-processed food ingredients are preferred (Figs 8A and 9A). Such ideal markers, however, may not always be available at high abundance due to dilution of food ingredients in complex foods or due to compound instability under the sometimes harsh chemical and physical conditions of food processing. In such cases, we see two options that are not mutually exclusive. (1) Chemical methods for the targeted enrichment of low abundant marker compounds, such as solid phase extractions, can be implemented for metabolite targeted food monitoring. Enrichment prior to chemical analysis may for example enable the use of rosmarinic acid and catechollactic acid for the verification of chia seeds in processed food. Demonstration of this option was beyond the scope of our current study. (2) As an alternative, chemical reaction products that are generated by the high temperature and sometimes high pressure or other extreme physico-chemical reaction conditions of food processing may become available as markers, e.g. succinic acid monomethylester, tyrosol, or 4-hydroxybenzaldehyde. Such reaction products may be applicable to specifically processed food material and may also validate the mode of food processing. Obviously chemical breakdown products can only be less specific of an ingredient than the more complex chemical structures that are present in non-processed materials. Nevertheless, such compounds have the potential to indicate normalcy of the added ingredient.

### On the use of metabolite patterns

We advocate, if available, the use of multiple marker substances and alternative rule sets to score seed identity or to create models of the presence and normalcy of ingredients in food material. We indicated such options of alternative rule sets by decision tree representations (Figs 3B and 5B). Single marker substances have a higher potential of being falsified or of being lost or diluted by food processing than more complex marker patters that comprise many compounds. In terms of preventing falsification we propose that markers may be used even though the exact chemical structure may not yet be elucidated. Non-identified or possibly non-disclosed marker substances are obviously more difficult to falsify. Non-identified compounds may still be used to diagnose the presence or normalcy of food ingredients, if the retention index and mass spectral properties are thoroughly documented, e.g. (Supplemental Data File S5), and if authenticated food reference materials that contain the non-identified compounds are available.

Clearly, our current study must be seen as an early stage and as a test case of marker search for seed identity. Such studies need to be extended, e.g. by testing marker stability under varying environments of seed production and storage, before applications in food declaration and regulation can be considered. We hope to have contributed to ongoing efforts to extend the tool box of metabolomics marker search but also reveal current limitations of the potential markers that we propose. For example, the rosmarinic acid biosynthesis pathway should be present in most Salvia species and beyond in other Lamiaceae. Therefore, the markers presented in this study may not yet be optimal. Diagnosis of seed integrity based on the current set of markers, rosmarinic acid and catechollactic acid, may not prevent exchange or falsification of chia seeds by seeds of other related species. Other metabolic markers may be present and become accessible by alternative technologies of chemical analysis, e.g. LC-MS. Also combination with the monitoring of DNA fingerprints must be considered. Respective integrated methods need to be optimized for our specific test case of seed ingredients and mode of food processing. Information of additional seed types, such as quinoa (*Chenopodium quinoa* Willd.), that were omitted from our current study, but may occur in similar food materials should extend the number of markers but may also restrict the specificity range of the currently available markers. Finally, alternative modes of food processing may generate additional chemical conversion products that may be monitored. For these reasons, we designed our study in process steps that can be iterated and extended towards additional materials or methods of chemical analysis. Most importantly, alternative methods of machine learning and feature extraction may become available or alternative statistical and variance-based methods may be seen worthwhile for testing feature extraction using our current data sets (Table S2, Table S4). We certainly did not explore the full scope of our data sets or compared more traditional and established approaches of metabolomic marker search, such as partial least squares to latent structures (PLS), orthogonal partial least squares projections to latent structures (O2PLS), or principal component analysis (PCA), that were reviewed, for example, by Fernandez and co-authors^49^.

## Methods and Materials

### Seed material and experimental bakery products

Authenticated seed batches of chia (*Salvia hispanica* L.), linseed (flax; *Linum usitatissimum* L.), and sesame (*Sesamum indicum* L.) that were sold for human consumption in local grocery stores or marketed via internet sources within the European Union were purchased in autumn 2016 in Berlin, Germany. Seed batches were authenticated by vendor information and in part by voluntary EU-regulated ECO labels (http://ec.europa.eu/environment/ecolabel/) of the acquired products. Seed morphology was visually checked for aberrant material or impurities, e.g. (Fig. 1A). The seed materials were named S_01 - S_28 and vendor information documented (Fig. 1; Table S1).

We produced experimental bakery products, i.e. wheat cookies, with 5, 10, 15, or 20% (w/w) defatted seed flour of single seed types. In addition, we generated wheat cookies with 10% (w/w) and 20% (w/w) of whole seeds. For the classification purpose of our study we combined these cookies throughout our current study into single classes that we labelled by 15 ± 5% (w/w) of whole seeds. Besides three wheat cookie classes that contained the separate whole seeds, we produced cookies with 15 ± 5% (w/w) of a mixture of whole seeds. The seed types in the mixture were in equal amounts, chia: linseed: sesame (1:1:1; (w/w/w). These seeds used for experimental cookie production were sold for human consumption in South America and bought from grocery stores in Córdoba, Argentina.

Partially defatted chia, sesame and flax flours were obtained according to the process described by Martínez and co-authors^52^ in a single baking campaign. Briefly, seeds were hydrated to 9.5% moisture, packed in air-tight bags, and stored for 48 h. The bags were shaken regularly to homogenize sample moisture. Hydrated seeds were conditioned to 60 °C and pressed using a screw press Komet (Model CA 59 G, IBG Monforts, Germany). Screw speed was 20 rpm. A 5 mm restriction was used. The meal obtained after oil extraction was subsequently ground with a coffee mill and passed through a 0.25 mm sieve. The milled and sieved fractions of defatted seeds were used for production of experimental cookies.

For the production of defatted flour cookies with increasing percentages of defatted seed flour the wheat flour was partially replaced by an equivalent amount of seed flour. A control formulation using only wheat flour was used to produce control cookies. In detail, wheat flour with or without the corresponding amount of replaced defatted seed flour, in total 45 g, were mixed to obtain Mix 1. For Mix 2, 20.2 g fat and 27 g powdered sugar were mixed separately with an electric mixer for 3 minutes. In a third container the additives 0.42 g sodium chloride, 0.50 g sodium bicarbonate and 2.25 g skimmed powdered milk were combined (Mix 3). Mix 2 and Mix 3 were mixed and stirred dry for 1 min using an electric mixer. Then, 4 mL water was added. Mixing continued for 1 min. Finally, Mix 1 was added. The complete combination of mixes was stirred for 2 min to obtain soft dough. The dough was stretched with a rolling pin and laminated to obtain a uniformly spread dough of 0.8 cm height. Cookies were cut by a metallic cutter of 45 mm inner diameter. The circular cookies were equally distributed on a baking plate with greaseproof paper and baked 11 min at 180 °C. Each procedure yielded 4 cookies per baking. Cookies with whole seeds were prepared as was described above by replacing the amount of wheat flour by whole seeds.

### Metabolome profiling of the volatile organic fraction (VOC)

Seed material was stored at −80 °C in 50 mL Falcon-tubes until measurement. For the analysis of whole seeds, 1 g ± 0.5% was aliquoted into 20 mL head-space-vials (Gerstel, Mülheim an der Ruhr, Germany). Analyses of 6 analytical replica per seed material (S_01 - S_28) were performed by solid phase micro extraction (SPME) and gas chromatography coupled to electron impact ionization/quadrupole mass spectrometry (GC-MS) using an Agilent 6890N24 gas chromatograph (Agilent Technologies, Böblingen, Germany; http://www.agilent.com) and a StableFlex^TM^ SPME-fiber with 65 µm polydimethylsiloxane/divinylbenzene (PDMS-DVB) coating (Supelco, Bellefonte, USA)^36,37^. Mass features that did not occur in 100% of all analytical replicates of a seed batch were set to “not detected” (Table S2). SPME samples were taken from the headspace with 10 min incubation at 45 °C, 5 min adsorption at 45 °C and 1 min desorption at 250 °C onto a DB-624 capillary column with 60 m length, 0.25 mm inner diameter, 1.40 μm film thickness (Agilent Technologies, Böblingen, Germany). The GC temperature programming was 2 min isothermal at 40 °C followed by a 10 °C/min ramping to 260 °C final temperature which was held constant for 10 min. The Agilent 5975B VL GC-MSD system was operated with a constant flow of helium at 1.0 mL/min. Desorption from the SPME fiber was at 16.6 psi with an initial 0.1 min pulsed-pressure at 25 psi. The subsequent purge was 1 min at a purge flow of 12.4 mL/min. GC-MS chromatograms were acquired with the mass range set to 30–300 mass to charge ratio (m/z) and a 20 Hz scan rate. Chromatography data files were visually controlled, exported as AIA-files resulting in NetCDF files format using Agilent ChemStation-Software and afterwards baseline-corrected with ChromaTOF Version 4.51.6.0 (LECO Instrumente GmbH, Mönchengladbach, Germany; http://www.leco.de) applying a method with settings of 1 for baseline offset, 9 for smoothing, 20 for peak width and 1 for signal to noise ratio S/N. Subsequent data processing was performed with all measured signal intensities, i.e. abundances, at threshold S/N ≥ 2 or at least 500 arbitrary abundance units. A standardized numerical data matrix was generated using TagFinder software and absolute time scale^43^. Signal abundances were normalized to seed weight (g, gram). Mass spectra were deconvoluted manually supervised using the Automated Deconvolution and Identification System (AMDIS) version 2.73 that was part of the National Institute of Standards and Technology (NIST) spectral search and analysis software (NIST17; https://www.nist.gov/srd/nist-standard-reference-database-1a-v17) or by ChromaTOF software. Compounds were annotated manually by mass spectral and retention index matching to the Golm Metabolome (GMD; http://gmd.mpimp-golm.mpg.de/)^53^ or to the NIST17 spectral databases.

### Metabolome profiling of the polar soluble fraction (POL)

Seed material that was stored at −80 °C in 50 mL plastic tubes Falcon-tubes was manually ground to powder under liquid nitrogen. The frozen powder was stored at −80 °C until further processing in 50 mL Falcon tubes. Analyses of 5 analytical replicates were performed per seed material (S_01 - S_28). Mass features that did not occur in 100% of all technical replicates of a seed batch were set to “not detected” (Table S2). Experimental bakery products were stored vacuum-packed at room-temperature for about 6 months until manual grinding of pooled material from 3–4 cookies of each bakery product under liquid nitrogen. The ground material was stored at −80 °C in 25 mL Falcon-tubes. Analyses of 4–5 analytical replicates per experimental bakery product were performed.

The extraction of both types of solid matrices was performed using 100 mg ± 5% material in 2.0 mL micro centrifuge tubes. Profiling of metabolites was performed as described previously^40,41^. Metabolites were extracted from frozen powder first by 360 µL methanol with 0.2 mg mL^−1^ U-^13^C-sorbitol (Sigma-Aldrich Chemie GmbH, Taufkirchen, Germany) added as internal standard. After 15 min at 70 °C we added 200 µL of chloroform and incubated 5 min at 37 °C. The liquid extract was partitioned into two phases by adding 400 µL bi-distilled H_2_O. Samples were thoroughly vortexed after adding water and phases separated by 5 min centrifugation at 14.000 rpm (equivalent to a centrifugal force of 20.8 g). Two aliquots of 160 µL from the upper fraction that is enriched in polar metabolites were retrieved after liquid phase extraction. Both aliquots of the polar phase (160 µL) were dried ~2 h at room temperature in a speed vacuum concentrator. One aliquot of the dried polar fraction was chemically derivatized by methoxyamination and trimethylsilylation. The other 160 µL aliquot was stored as a backup sample at −80 °C. A mixture of n-alkanes, C_10_, C_12_, C_15_, C_18_, C_19_, C_22_, C_28_, C_32_ and C_36_, (Sigma-Aldrich Chemie GmbH, Taufkirchen, Germany) served as retention index standards. A 1 µL aliquot of the derivatized samples was injected in splitless mode at 230 °C into a 6890N24 gas chromatograph (Agilent Technologies, Böblingen, Germany; http://www.agilent.com). The injected material was separated on a Varian FactorFour column (VF-5ms, length 30 m, diameter 0.25 mm, and 0.25 µm film thickness (Agilent Technologies, Böblingen, Germany) using the following temperature programme: 1 min at 70 °C, ramp to 350 °C at 9°/min, 5 min at 350 °C, and reset to initial temperature. Compounds were detected by electron ionization/time-of-flight mass spectrometry (GC-EI-TOF-MS) using a Pegasus III TOF mass spectrometer (LECO Instrumente GmbH, Möchengladbach, Germany). Chromatograms were obtained and baseline corrected after visual quality assessment by ChromaTOF software as described above but applying a method with settings of 1 for baseline offset, 5 for smoothing, 10 for peak width and 1 for S/N. Data matrices were generated by using all measured signal abundances at S/N ≥ 2 or at least 150 abundance units. The numerical data matrices including retention index (RI) calculation of each sample were generated in a standardized manner using TagFinder software. Initial signal abundances were normalized to sample weight (mg) and the internal standard U-^13^C-sorbitol using the abundance of the U-^13^C-sorbitol specific mass fragment m/z = 323.

### Metabolome profiling of the components of the solid fraction after hydrolysis (SOL)

The components of the solid fraction were analyzed according to a prior publication^42^. The responses of mass features were averaged across 2–5 analytical replicates. Mass features that did not occur in 100% of all technical replicates of a seed batch were set to “not detected” (Table S2). In detail, after extraction of the polar soluble fraction, i.e. the removal of two times 160 µL of the polar phase, the remaining pellet was washed twice with 1.5 mL of MeOH:CHCl_3_:H_2_O (2.5:1:1, v/v/v). During the washes the remaining pellet and solids were transferred to 2 mL micro tubes with screw-caps (Ref No. 72.693; Sarstedt, Nürnbrech, Germany). The complete liquid supernatant was removed after centrifugation 5 min at 14.000 rpm equivalent to a centrifugal force of 20.8 g. After supernatant removal, the final pellet was dried in a speed vacuum concentrator 2 days at room temperature. The dry pellet was stored at −80 °C under nitrogen until further analysis. Hydrolysis was performed by 300 µL of a 2 M trifluoroacetic acid (TFA) solution in H_2_O, which was added to the still frozen pellet. Incubation was 5 h at 121 °C in a heated shaker. A small hole in the lid of the tube enabled pressure equilibration without complete evaporation of the TFA solution. After cooling to room temperature 200 µL of 2-propanol was added and the material dried 2 h at room temperature in a speed vacuum concentrator. This step was repeated twice. We finally added 300 µL of bi-distilled H_2_O, thoroughly re-suspended and vortexed the material and centrifuged 5 min at 14.000 rpm (equivalent to a centrifugal force of 20.8 g). An aliquot of 200 µL from this final supernatant was dried in a 1.5 mL Eppendorf tube using a speed vacuum concentrator. The dried fraction contained hydrolysed components of the solid polymers and was chemically derivatized and analyzed by GC-MS exactly as described above for the polar soluble fraction. The method includes carbohydrate monomers and non-carbohydrate cell wall components. The initially recorded signal abundances were normalized to the sample weight (mg) and the abundance of n-dotriacontane (Sigma-Aldrich Chemie GmbH, Taufkirchen, Germany) using compound specific mass fragment m/z = 85. The internal standard was added prior to chemical derivatization and after the last vacuum concentration step.

### Mass feature selection using random forests

In this study we choose to apply mass feature selection by random forest (RF) technology using the data matrices of normalized mass feature abundances. Non-detected, missing data within the data matrices, i.e. missing values, were replaced by estimates of the respective detection limits, i.e. the minimum value after normalization across all mass features and samples (Tables S2 and S4). Prior to RF analyses we removed all mass features with less than 10 observations across the complete set of samples and omitted mass features that were not observed in 100% of all replicates of one sample type.

RFs are a variant of machine learning methods. RFs use unpruned decision trees that are built on a bootstrap sample of the training data using a randomly selected subset of predictors^46^. The R-package randomForest^45^ was used to train classification models of the identity of non-processed seeds or the identity of seed material that was added to wheat cookies. Each RF model had 10^4^ trees with up to 500 variables tested for each node. We evaluated the confusion matrices of each trained classification model and for variable selection, the mean decrease of accuracy measures and the respective mean decrease of Gini index that is equivalent to tree node purity. Variables of RF analyses in this study were the recorded mass features of GC-MS profiles. We averaged the mean decrease of Gini index and the mean decrease of accuracy measures of the top-ranking mass features across repeated classification models and calculated standard errors. Linear correlation between the two variable importance measures, mean decrease of accuracy and mean decrease of Gini index, was calculated by r² of a Pearson’s correlation coefficient.

#### Classification models of three non-processed seed types

For the classification models of the non-processed seed types, chia, linseed or sesame, we pre-selected mass features that were 1-way ANOVA (P < 10^−5^) significant for the factor seed type. We reduced the redundancy of mass features by selecting the most significant feature with least missing values among the groups of co-eluting mass features. Note that compounds are typically represented in GC-MS profiles by multiple mass features that are generated by electron impact ionization and fragmentation. Mass features of the same compound may have different signal to noise values and selectivity depending on the fragmentation reactions and on the sample-specific co-eluting compounds. We created 10 classification models that were all trained to classify the three seed classes. For each repeated classification model we selected randomly a different set of 14 training profiles out of the 28 metabolite profiles from the different non-processed seeds. According to their confusion matrices of all 10 trained models classified the different non-processed seed types without error. The details of these confusion matrices were therefore not further analysed. The selected mass features were ranked according to averaged mean decrease of accuracy.

#### Classification models of four experimental cookie types

Pre-selection of mass features by ANOVA or removal of redundant mass features that represented the same compound was non-mandatory for the RF feature selection process of the randomForest R-package and omitted in this application case. We defined four classes of cookies irrespective of the amount of added seed material and irrespective of seed pre-processing. The four classes were (1) experimental wheat cookies without additions, and cookies with additions of (2) chia, (3) linseed, or (4) sesame seed irrespective of either added whole seeds or of added seed flour material. The cookies with equal mixtures of three seed types were not included.

In a first round of feature selection we created a model that was based on the matrix of all available mass features using random samplings of 46 training wheat cookie profiles of recorded mass features out of a total of 93 profiles (Table S4, columns D-CR). We selected the top important mass feature according to mean decrease of accuracy and all redundant mass features from the top 30 that represented the same compound. These mass features were omitted from the next round of feature selection. In the second and all subsequent rounds the selected top mass features and their redundant mass features were excluded from subsequent RF analysis rounds. We iterated this take-the-top variable-out approach^12^ twelve times until the overall error of the trained models exceeded 33%.

Finally we created a subset matrix that contained all previously selected 84 mass features including all redundant mass features. We evaluated and ranked these mass features by again creating RF classification models of four experimental cookie types but based only on the selected subset. For this purpose we created 12 classification models by repeated random selection of 46 training profiles out of 93 profiles. Resulting mean decrease of accuracy, mean decrease of Gini index of the mass features were averaged and features ranked as described above. The 12 classification models for the classification of the presence or absence of different seed material in wheat cookies had variable overall errors depending on the sampled subset of training profiles. To characterize overall errors and respective variance of the models we averaged results of the confusion matrices. We calculated an average overall error and average class false negative rates or class false discovery rates. Besides averages we report respective maximal false negative rates and false discovery rates of the 12 sampled classification models.

To test the chosen iterative feature selection process we added selected mass features to the final matrix of 84. We added six additional mass features that represented metabolites that were relevant for the classification models of three non-processed seed types and added two additional mass features. The normalized abundances of these two mass features were correlated to the amount of added seed flour but were not chosen by our RF based approach. Correlation between normalized abundance and amount of added seed flour was determined using the Pearson’s correlation coefficient.

### Mass feature selection using Min/Max ratios

As an alternative mass feature selection for the classification of non-processed seed types, we calculated ratios of minimum (Min) normalized abundances of mass features from one seed type over the maximum (Max) normalized abundance that was observed in the other two seed types, namely the ratios Min_Chia_/Max_Linseed, Sesame_, Min_Linseed_/Max_Chia, Sesame_, and Min_Sesame_/Max_Chia, Linseed_. Missing values were substituted before ratio-calculations by an estimate of the detection limit. Prior data processing was as described above. The ratios were used as a simple algorithmic solution to the selection of potential “positive” markers for the presence of a seed type. We selected the top 15 metabolites, i.e. non-redundant mass features, according to the largest ratios of mass features across the three variants of ratio calculations (Table S2, columns AF-AT).

### Data visualization and pre-processing

Heat maps and ANOVA were created by the multi-experiment viewer software, MeV (Version 4.9.0; https://sourceforge.net/projects/mev-tm4/)^54^ and the Microsoft-Excel 2010 program. ANOVA was used in the case of marker discovery of non-processed seeds for ranking mass features prior to RF based feature extraction. ANOVA was not used for feature extraction and subsequently discarded as means of pre-filtering. For the purpose of ranking, we did not test for normality, homoscedasticity prior to ANOVA or applied multiple-testing-correction. Principal component analyses (PCA) were performed using log_10_-transformed abundance ratios relative to the median across all samples without scaling. PCA was intended as a first orientation. Missing value replacement by an estimate of the detection limits of each analytical method and data pre-processing were kept consistent with Min/Max ratio calculations. PCA calculations were via the “stats” R Package and the MetaGeneAlyse web application (Version 1.7.1; http://metagenealyse.mpimp-golm.mpg.de). Student´s *t*-tests were calculated by the Microsoft-Excel 2010 program. Tukey´s test using a generalized linear model was calculated by the R Package “multcompView”.

## Supplementary information

Table S1

Table S2

Table S3

Table S4

Data file S5
