# Supplementary material for: Discovery of food identity markers by metabolomics and machine learning technology
Source: Sci Rep. 2019 Jul 4;9:9697. doi: 10.1038/s41598-019-46113-y (PMC6609671; doi:10.1038/s41598-019-46113-y)
Supplement: Supplementary file 5 — Data file S5 [file 41598_2019_46113_MOESM5_ESM.docx]

Name: C01_POL_1175.17_189

Synon: Metabolite name: Monomethyl succinate

Synon: Analyte name: M001706_A117010-101-xxx_NA_1,168.56_PRED_VAR5_ALK_Succinic acid methylester (1TMS)

Synon: Analyte name: Monomethyl succinate, trimethylsilyl ester

Synon: RI: 1175 iu

Synon: Formula: C8H16O4Si

Synon: MW: 204 Exact Mass: 204.081785 CAS#: 86827-76-1

DB#: 54

Num Peaks: 295

73 387; 75 304; 76 19; 79 21; 89 999;

90 64; 91 31; 93 3; 94 1; 101 3;

105 35; 106 2; 107 5; 108 2; 109 1;

110 1; 114 68; 115 49; 121 1; 122 2;

123 5; 128 2; 129 101; 130 2; 133 1;

145 2; 153 2; 156 3; 157 17; 158 4;

162 2; 168 3; 169 11; 172 5; 173 60;

174 27; 175 5; 176 1; 182 2; 187 1;

189 205; 190 26; 191 10; 192 3; 196 1;

198 2; 202 1; 203 1; 206 2; 207 2;

210 2; 211 1; 212 1; 216 2; 217 7;

218 3; 219 2; 220 2; 222 5; 223 3;

224 1; 228 3; 229 2; 230 2; 231 3;

232 2; 235 3; 236 1; 237 3; 238 3;

239 2; 240 1; 242 2; 244 2; 245 1;

246 4; 248 2; 249 1; 251 4; 252 1;

253 1; 254 1; 256 1; 257 1; 259 2;

261 2; 262 1; 263 2; 264 1; 265 1;

266 5; 267 2; 268 3; 269 3; 270 3;

271 10; 272 4; 273 4; 274 3; 275 1;

276 1; 277 2; 278 2; 279 1; 280 1;

281 1; 282 2; 283 1; 284 6; 285 2;

286 2; 287 1; 288 1; 289 5; 290 4;

292 3; 294 1; 295 2; 297 3; 298 1;

300 2; 301 2; 302 3; 304 5; 305 4;

306 1; 307 5; 308 2; 309 3; 311 5;

314 1; 315 3; 316 1; 317 5; 318 2;

320 1; 321 1; 322 2; 323 1; 325 4;

326 1; 327 2; 328 2; 329 5; 331 2;

332 3; 333 1; 336 2; 337 2; 338 2;

339 2; 340 2; 341 5; 343 1; 344 1;

347 2; 348 1; 350 2; 351 3; 352 4;

353 3; 354 4; 355 1; 356 3; 357 3;

358 1; 359 2; 360 5; 361 2; 362 2;

363 7; 365 2; 366 3; 367 3; 368 3;

369 3; 370 3; 371 3; 372 3; 373 3;

375 4; 379 3; 380 2; 381 1; 382 2;

383 1; 384 2; 385 1; 386 1; 389 1;

390 3; 392 3; 393 4; 395 3; 397 2;

399 1; 400 3; 402 3; 403 3; 404 1;

405 2; 406 3; 408 3; 409 5; 410 1;

413 2; 414 1; 415 3; 416 3; 417 2;

418 2; 421 1; 423 1; 424 1; 427 2;

428 4; 429 1; 430 2; 432 3; 435 2;

436 2; 437 4; 439 4; 440 1; 442 1;

443 1; 445 2; 447 3; 448 2; 449 1;

450 2; 453 2; 456 3; 458 2; 460 2;

461 2; 462 2; 463 2; 464 2; 466 1;

474 3; 478 1; 479 1; 480 1; 482 3;

483 2; 484 3; 487 1; 488 3; 493 2;

494 1; 497 4; 498 1; 499 1; 501 2;

503 3; 504 3; 505 2; 508 1; 509 1;

512 4; 513 5; 515 1; 517 1; 519 2;

520 2; 525 1; 527 4; 528 3; 530 1;

531 1; 532 1; 534 4; 536 1; 541 2;

542 1; 543 1; 544 2; 545 2; 547 2;

552 1; 554 3; 557 3; 558 3; 560 2;

563 2; 566 1; 568 1; 569 3; 573 3;

574 1; 577 1; 586 1; 590 2; 591 2;

Name: C02_POL_1379.80_141

Synon: Metabolite name: non-identified

Synon: RI: 1380 iu

DB#: 48

Num Peaks: 107

70 4; 72 13; 73 555; 74 45; 75 509;

76 30; 77 30; 81 12; 83 272; 84 2;

85 330; 86 11; 87 35; 89 999; 90 75;

91 40; 93 2; 97 4; 99 3; 101 30;

105 296; 106 6; 107 13; 109 158; 110 1;

111 8; 112 13; 113 123; 114 2; 115 72;

116 25; 121 119; 122 49; 131 5; 139 4;

140 8; 141 621; 142 24; 143 58; 153 2;

157 38; 170 6; 173 189; 174 26; 175 4;

183 16; 189 118; 190 3; 199 19; 200 1;

203 1; 205 21; 206 3; 212 1; 215 128;

216 32; 217 31; 220 16; 226 4; 240 8;

243 2; 244 1; 247 52; 248 10; 253 13;

255 12; 260 1; 265 11; 266 1; 280 10;

288 5; 296 16; 297 6; 300 3; 306 10;

311 3; 312 4; 315 11; 318 7; 329 5;

331 5; 335 24; 336 25; 342 2; 346 2;

348 1; 355 3; 362 6; 364 5; 367 7;

392 4; 399 1; 402 2; 414 1; 426 8;

441 1; 453 7; 471 4; 478 14; 481 4;

486 2; 488 7; 494 5; 523 6; 545 1;

556 1; 590 2;

Name: C04_POL_1497.34_208

Synon: Metabolite name: 4-Hydroxybenzaldehyde

Synon: Analyte name: M001447_A150014-101-xxx_NA_1,493.64_PRED_VAR5_ALK_4-Hydroxybenzaldehyde (1MeOX) (1TMS) MP

Synon: Analyte name: 4-Hydroxybenzaldehyde (1MeOX) (1TMS)

Synon: RI: 1497 iu

Synon: Formula: C11H17NO2Si

Synon: MW: 223 Exact Mass: 223,10286

DB#: 86

Num Peaks: 77

76 24; 88 68; 89 107; 90 121; 91 343;

92 96; 93 98; 94 154; 102 54; 104 129;

107 64; 108 85; 109 13; 120 71; 121 82;

122 33; 123 69; 132 25; 135 208; 136 89;

137 54; 138 28; 150 217; 151 99; 152 75;

158 50; 159 37; 160 90; 162 54; 163 17;

164 46; 165 104; 166 98; 167 71; 171 5;

172 32; 173 27; 174 24; 176 882; 177 640;

178 206; 180 36; 181 70; 185 37; 186 31;

187 35; 188 7; 190 17; 192 150; 193 93;

194 73; 195 78; 196 61; 197 49; 198 27;

199 47; 200 57; 201 15; 202 44; 203 28;

204 46; 205 38; 206 47; 207 30; 208 778;

209 146; 210 28; 211 53; 215 47; 216 87;

220 22; 222 34; 223 999; 224 199; 225 104;

226 19; 227 4;

Name: C05_POL_1684.05_103

Synon: Metabolite name: non-identified

Synon: Analyte name: M000000_A170001-101-xxx_NA_1,688.90_PRED_VAR5_ALK_NA170001 (classified unknown)

Synon: Note: similar to pentitol (5TMS)

Synon: RI: 1684 iu

DB#: 9

Num Peaks: 137

70 2; 71 1; 72 17; 73 999; 74 85;

75 69; 76 3; 77 2; 81 9; 82 1;

83 4; 84 1; 85 2; 86 1; 87 5;

88 4; 89 23; 90 2; 91 1; 97 1;

98 1; 99 2; 100 1; 101 21; 102 3;

103 295; 104 30; 105 13; 106 1; 111 2;

113 4; 114 1; 115 5; 116 10; 117 100;

118 10; 119 8; 120 1; 127 1; 128 1;

129 123; 130 16; 131 27; 132 3; 133 54;

134 7; 135 4; 137 1; 141 1; 142 1;

143 8; 144 1; 145 3; 146 1; 147 253;

148 44; 149 28; 150 3; 151 1; 153 1;

155 3; 156 1; 157 18; 158 2; 159 2;

160 2; 161 3; 163 3; 164 1; 165 1;

169 1; 170 1; 171 2; 172 1; 173 1;

175 5; 176 1; 177 4; 178 1; 179 1;

187 1; 189 42; 190 9; 191 29; 192 5;

193 2; 201 1; 203 16; 204 46; 205 104;

206 22; 207 11; 208 1; 215 1; 216 1;

217 263; 218 68; 219 29; 220 4; 221 6;

222 1; 223 1; 229 5; 230 1; 231 1;

242 1; 243 18; 244 4; 245 2; 246 1;

247 1; 277 13; 278 4; 279 2; 280 1;

291 3; 292 1; 293 1; 305 2; 306 5;

307 50; 308 14; 309 6; 310 1; 317 5;

318 3; 319 42; 320 11; 321 5; 322 1;

332 4; 333 2; 334 1; 395 1; 407 1;

422 1; 423 1;

Name: C06A_POL_2210.58_337

Synon: Metabolite name: Linoleic acid

Synon: Analyte name: M000488_A221003-101-xxx_NA_2,209.91_TRUE_VAR5_ALK_Octadecadienoic acid, 9,12-(Z,Z)- (1TMS)

Synon: Analyte name: 9,12-Octadecadienoic acid (Z,Z)-, TMS derivative

Synon: RI: 2211 iu

Synon: Formula: C21H40O2Si

Synon: MW: 352 Exact Mass: 352.279757 CAS#: 56259-07-5

DB#: 11

Num Peaks: 210

70 29; 71 15; 72 21; 73 772; 74 99;

75 999; 76 41; 77 167; 78 60; 79 334;

80 275; 81 617; 82 287; 83 139; 85 18;

87 6; 88 13; 89 53; 90 6; 91 109;

92 35; 93 209; 94 163; 95 385; 96 185;

97 87; 99 20; 100 2; 101 12; 102 8;

103 16; 104 4; 105 71; 106 25; 107 136;

108 85; 109 132; 110 120; 111 60; 115 6;

116 27; 117 112; 118 25; 119 23; 120 21;

121 146; 122 70; 123 75; 124 50; 125 30;

126 5; 128 5; 129 169; 130 15; 131 105;

132 31; 133 24; 134 17; 135 134; 136 109;

137 31; 138 20; 139 29; 141 12; 142 3;

143 12; 144 5; 145 29; 146 6; 147 39;

148 13; 149 78; 150 142; 151 25; 152 7;

153 7; 154 6; 155 15; 156 9; 157 23;

159 27; 160 4; 161 8; 162 2; 163 33;

164 60; 171 17; 172 7; 173 28; 175 6;

177 15; 178 67; 179 15; 180 4; 183 15;

187 11; 189 6; 191 18; 192 5; 193 5;

200 7; 201 13; 202 6; 204 20; 205 10;

206 2; 208 7; 209 2; 210 1; 211 1;

214 1; 215 14; 216 2; 217 9; 218 8;

219 8; 220 44; 221 2; 224 1; 225 4;

226 3; 227 8; 228 1; 229 9; 231 1;

232 2; 233 1; 234 9; 235 3; 239 4;

240 1; 241 1; 242 1; 243 9; 244 2;

247 2; 248 1; 249 1; 251 2; 254 1;

255 4; 259 1; 262 93; 263 17; 272 3;

276 1; 282 1; 285 1; 290 1; 296 3;

304 1; 310 2; 313 1; 315 1; 320 1;

327 1; 336 9; 337 80; 338 31; 343 2;

350 1; 352 6; 362 2; 364 2; 366 1;

368 2; 369 2; 371 1; 374 1; 377 1;

378 1; 380 1; 381 1; 383 1; 385 1;

393 1; 394 1; 396 1; 404 1; 407 1;

413 1; 414 1; 415 1; 417 1; 420 1;

421 1; 429 1; 433 1; 435 1; 437 5;

438 1; 451 1; 453 1; 456 1; 458 1;

471 1; 476 1; 504 1; 511 1; 535 1;

547 1; 563 1; 567 1; 568 1; 572 1;

580 1; 594 1; 595 1; 598 1; 600 1;

Name: C06B_POL_2215.16_339

Synon: Metabolite name: Oleic Acid

Synon: Analyte name: M000486_A222001-101-xxx_NA_2,221.65_TRUE_VAR5_ALK_Octadecenoic acid, 9-(Z)- (1TMS)

Synon: Analyte name: Oleic Acid, (Z)-, TMS derivative

Synon: RI: 2215 iu

Synon: Formula: C21H42O2Si

Synon: MW: 354 Exact Mass: 354.295406 CAS#: 21556-26-3

DB#: 14

Num Peaks: 314

70 50; 71 33; 72 50; 73 841; 74 88;

75 999; 76 77; 77 80; 78 20; 79 119;

80 58; 81 269; 82 136; 83 165; 84 193;

85 41; 86 14; 87 10; 88 10; 89 30;

90 8; 91 41; 92 17; 93 75; 94 45;

95 179; 96 229; 97 146; 98 159; 99 38;

100 3; 101 12; 102 1; 104 2; 105 34;

106 10; 107 36; 108 22; 109 95; 110 92;

111 73; 112 28; 113 6; 114 2; 115 7;

116 63; 117 585; 118 65; 119 53; 120 13;

121 54; 122 18; 123 70; 124 46; 125 27;

126 6; 127 7; 128 6; 129 432; 130 65;

131 105; 132 121; 133 50; 134 28; 135 38;

136 21; 137 43; 138 30; 139 15; 140 2;

141 7; 142 8; 143 34; 144 4; 145 180;

146 28; 147 23; 148 15; 149 21; 150 22;

151 28; 152 31; 153 8; 154 2; 155 21;

156 6; 157 19; 158 4; 159 20; 160 2;

161 6; 162 4; 163 4; 164 12; 165 15;

166 20; 167 9; 168 6; 169 15; 170 6;

171 29; 172 14; 173 11; 174 6; 175 6;

176 3; 177 2; 178 6; 179 7; 180 34;

181 5; 182 2; 183 19; 184 5; 185 51;

186 11; 187 13; 188 7; 189 5; 190 4;

191 5; 193 4; 194 4; 195 4; 197 4;

198 1; 199 60; 200 7; 201 15; 202 1;

203 5; 204 3; 206 4; 207 5; 208 3;

209 2; 210 2; 211 6; 212 1; 213 9;

215 4; 216 1; 219 7; 220 10; 221 8;

222 32; 223 7; 225 4; 227 11; 228 3;

229 2; 230 1; 234 4; 235 6; 236 6;

239 3; 240 2; 241 9; 242 3; 243 3;

244 1; 245 2; 246 1; 248 1; 249 2;

251 1; 253 3; 254 1; 255 5; 256 2;

257 7; 258 2; 260 1; 262 4; 263 2;

264 28; 265 5; 266 1; 269 1; 271 3;

272 3; 273 2; 275 1; 277 1; 278 1;

279 1; 280 2; 282 1; 285 1; 292 2;

294 2; 295 4; 296 2; 297 1; 298 1;

299 3; 300 2; 302 1; 303 1; 304 1;

305 1; 306 2; 307 2; 308 1; 311 1;

315 1; 316 1; 317 1; 318 2; 319 1;

320 2; 321 3; 322 2; 323 1; 325 2;

326 1; 327 1; 328 1; 329 1; 330 3;

332 1; 333 2; 334 1; 336 3; 337 12;

338 14; 339 115; 340 37; 341 8; 342 4;

343 1; 345 1; 349 1; 352 4; 353 3;

354 7; 355 3; 356 3; 358 1; 359 1;

360 3; 361 5; 364 2; 367 1; 368 2;

370 1; 373 1; 374 2; 376 1; 379 1;

381 1; 382 1; 383 1; 384 1; 392 1;

395 1; 399 1; 400 1; 402 1; 404 1;

405 1; 407 1; 409 1; 410 1; 411 1;

413 1; 414 1; 423 1; 428 1; 429 1;

436 1; 438 1; 439 1; 444 1; 452 1;

454 1; 455 1; 457 1; 458 1; 459 1;

462 1; 464 1; 469 1; 473 1; 474 1;

484 1; 488 1; 489 1; 491 1; 495 1;

499 1; 511 1; 515 1; 517 1; 523 1;

531 1; 532 1; 535 1; 538 1; 541 1;

551 1; 554 1; 555 1; 556 1; 558 1;

559 1; 561 1; 566 1; 575 1; 578 1;

582 1; 585 1; 591 1; 593 1;

Name: C07_POL_2960.08_433

Synon: Metabolite name: Galactinol

Synon: Analyte name: M000673_A299002-101-xxx_NA_2,966.29_TRUE_VAR5_ALK_Galactinol (9TMS)

Synon: Analyte name: Galactinol, nonakis(trimethylsilyl) ether

Synon: RI: 2960 iu

Synon: Formula: C39H94O11Si9

Synon: MW: 990 Exact Mass: 990.471954

DB#: 18

Num Peaks: 198

70 2; 71 2; 72 9; 73 999; 74 83;

75 69; 76 3; 77 3; 79 1; 81 20;

82 2; 83 4; 84 1; 85 4; 86 1;

87 4; 88 1; 89 5; 90 1; 91 1;

95 1; 97 3; 98 1; 99 4; 100 1;

101 12; 102 3; 103 155; 104 16; 105 7;

106 1; 109 5; 111 3; 112 1; 113 6;

114 1; 115 4; 116 6; 117 20; 118 2;

119 3; 125 1; 126 1; 127 4; 128 1;

129 146; 130 20; 131 24; 132 3; 133 35;

134 5; 135 3; 139 2; 140 1; 141 3;

142 4; 143 25; 144 3; 145 5; 146 1;

147 225; 148 36; 149 26; 150 2; 151 2;

153 3; 154 1; 155 11; 156 3; 157 14;

158 2; 159 2; 160 1; 161 4; 162 1;

163 3; 164 1; 167 1; 169 39; 170 6;

171 4; 173 4; 174 1; 175 3; 177 5;

178 1; 179 1; 181 1; 183 1; 185 1;

187 1; 189 19; 190 9; 191 171; 192 32;

193 16; 194 1; 195 1; 199 1; 201 2;

202 1; 203 9; 204 640; 205 128; 206 55;

207 11; 208 2; 215 3; 216 2; 217 207;

218 51; 219 23; 220 3; 221 14; 222 3;

223 1; 227 2; 228 1; 229 6; 230 19;

231 10; 232 3; 233 3; 234 1; 235 1;

239 1; 241 2; 242 1; 243 23; 244 6;

245 7; 246 2; 247 1; 255 2; 256 1;

257 2; 258 1; 259 2; 263 1; 265 9;

266 2; 267 1; 270 1; 271 16; 272 4;

273 2; 274 1; 278 1; 279 1; 289 1;

290 1; 291 7; 292 1; 293 7; 294 2;

295 1; 303 1; 304 6; 305 35; 306 14;

307 6; 308 1; 317 3; 318 11; 319 14;

320 5; 321 2; 330 1; 331 5; 332 3;

333 1; 342 1; 343 15; 344 5; 345 4;

346 1; 347 1; 359 2; 360 3; 361 31;

362 12; 363 5; 364 1; 393 1; 419 1;

420 1; 432 3; 433 25; 434 11; 435 6;

436 2; 437 1; 451 1; 507 1; 508 1;

523 1; 524 1; 539 1;

Name: C08_POL_3345.95_129

Synon: Metabolite name: Raffinose

Synon: Analyte name: M000049_A337002-101-xxx_NA_3,350.64_TRUE_VAR5_ALK_Raffinose (11TMS)

Synon: Analyte name: Raffinose (11TMS)

Synon: RI: 3346 iu

Synon: Formula: C51H120O16Si11

Synon: MW: 1296 Exact Mass: 1296.60383

DB#: 8

Num Peaks: 234

70 2; 71 4; 72 9; 73 999; 74 79;

75 78; 76 5; 77 4; 78 1; 80 1;

81 30; 82 2; 83 7; 84 1; 85 9;

86 1; 87 4; 88 4; 89 11; 91 2;

92 1; 95 1; 96 1; 97 4; 98 2;

99 4; 101 17; 102 6; 103 155; 104 16;

105 8; 106 1; 107 1; 109 11; 111 4;

112 1; 113 7; 114 1; 115 8; 116 8;

117 43; 118 3; 119 3; 124 1; 125 1;

126 1; 127 4; 128 2; 129 175; 130 24;

131 26; 132 2; 133 34; 134 4; 135 3;

137 1; 139 4; 140 1; 141 7; 142 6;

143 26; 144 3; 145 6; 146 2; 147 161;

148 28; 149 23; 150 2; 151 2; 152 1;

153 4; 154 1; 155 24; 156 4; 157 27;

158 3; 159 2; 161 4; 163 5; 165 1;

167 1; 169 125; 170 16; 171 13; 172 2;

173 3; 175 4; 177 4; 179 1; 181 2;

182 1; 183 7; 185 2; 187 1; 188 1;

189 26; 190 6; 191 53; 192 9; 193 5;

195 1; 197 2; 198 1; 199 4; 200 2;

201 2; 202 1; 203 13; 204 169; 205 49;

206 15; 207 8; 210 1; 211 1; 213 1;

214 1; 215 6; 216 2; 217 183; 218 50;

219 21; 220 4; 221 8; 222 2; 223 1;

227 3; 228 1; 229 9; 230 14; 231 11;

232 2; 233 2; 234 1; 235 1; 236 1;

240 1; 241 2; 242 2; 243 48; 244 12;

245 12; 246 4; 247 3; 248 1; 249 1;

250 1; 252 1; 254 1; 255 1; 257 11;

258 3; 259 5; 260 1; 261 1; 264 1;

265 1; 269 1; 270 2; 271 50; 272 12;

273 7; 274 1; 276 1; 277 2; 279 1;

282 1; 285 1; 289 3; 290 2; 291 5;

292 2; 293 2; 294 1; 295 1; 301 1;

303 1; 304 2; 305 6; 306 4; 307 2;

313 1; 317 3; 318 2; 319 15; 320 7;

321 3; 322 1; 323 1; 326 1; 327 1;

328 1; 330 1; 331 6; 332 4; 333 4;

334 1; 335 1; 336 1; 337 1; 338 1;

345 2; 347 1; 348 1; 349 1; 352 1;

353 1; 360 18; 361 229; 362 86; 363 46;

364 9; 365 4; 366 1; 368 1; 376 1;

378 1; 391 1; 414 1; 423 1; 435 1;

436 5; 437 37; 438 17; 439 8; 440 2;

447 1; 450 4; 451 23; 452 11; 453 4;

469 1; 560 1; 593 1; 594 1;

Name: C09_POL_3426.44_272

Synon: Metabolite name: non-identified

Synon: Note: similar to melezitose (11TMS)

Synon: RI: 3426 iu

DB#: 11

Num Peaks: 247

70 4; 71 4; 72 8; 73 999; 74 90;

75 101; 76 5; 77 8; 78 1; 79 1;

80 1; 81 28; 82 2; 83 7; 84 1;

85 8; 86 1; 87 4; 88 5; 89 11;

90 1; 91 1; 94 1; 95 2; 97 6;

98 1; 99 6; 100 1; 101 17; 102 4;

103 179; 104 18; 105 8; 107 1; 109 13;

110 1; 111 4; 112 1; 113 7; 114 1;

115 7; 116 6; 117 43; 118 5; 119 5;

125 1; 127 5; 128 2; 129 190; 130 27;

131 29; 132 2; 133 39; 134 5; 135 2;

137 1; 139 4; 140 2; 141 4; 142 7;

143 30; 144 3; 145 8; 146 1; 147 174;

148 29; 149 25; 150 3; 151 1; 153 5;

154 1; 155 30; 156 5; 157 33; 158 4;

159 3; 161 5; 163 4; 167 2; 168 1;

169 145; 170 20; 171 12; 172 2; 173 6;

174 1; 175 3; 177 4; 181 3; 182 2;

183 8; 184 2; 185 2; 187 2; 189 30;

190 7; 191 68; 192 10; 193 6; 195 2;

197 2; 199 6; 200 1; 201 2; 202 2;

203 11; 204 253; 205 67; 206 20; 207 9;

208 1; 209 1; 215 5; 216 3; 217 186;

218 49; 219 21; 220 4; 221 7; 222 2;

223 2; 227 3; 228 2; 229 10; 230 25;

231 18; 232 6; 233 6; 234 1; 235 1;

236 1; 240 1; 241 4; 242 1; 243 51;

244 13; 245 12; 246 2; 247 8; 248 2;

249 1; 251 1; 255 2; 256 1; 257 6;

258 2; 259 5; 260 1; 261 1; 263 1;

265 1; 267 1; 269 1; 270 3; 271 90;

272 21; 273 12; 274 2; 275 2; 277 1;

278 1; 279 1; 280 1; 282 1; 283 1;

285 1; 287 1; 288 1; 289 2; 290 1;

291 6; 292 3; 293 3; 294 1; 301 1;

303 1; 304 2; 305 7; 306 4; 307 3;

308 1; 317 5; 318 3; 319 15; 320 7;

321 2; 322 1; 324 1; 329 1; 330 1;

331 11; 332 5; 333 3; 334 1; 335 1;

336 1; 339 1; 344 1; 345 3; 346 2;

347 1; 348 1; 349 1; 350 1; 351 1;

352 1; 359 1; 360 16; 361 225; 362 92;

363 46; 364 9; 365 3; 372 1; 374 1;

375 1; 377 1; 393 2; 394 1; 407 1;

434 1; 438 1; 450 2; 451 9; 452 4;

453 1; 454 1; 462 1; 472 1; 482 1;

487 1; 489 1; 490 1; 500 1; 521 1;

523 1; 532 1; 541 1; 542 1; 559 1;

561 1; 569 1; 570 1; 576 1; 577 1;

581 1; 600 1;

Name: C10_POL_1335.52_97

Synon: Metabolite name: non-identified

Synon: Note: similar to itaconic acid (2TMS)

Synon: RI: 1336 iu

DB#: 59

Num Peaks: 212

70 22; 72 12; 73 523; 75 128; 81 7;

82 10; 83 5; 86 5; 87 3; 89 7;

90 2; 97 999; 98 64; 99 10; 100 9;

103 7; 112 4; 113 4; 118 1; 127 3;

128 2; 130 5; 131 29; 133 21; 135 1;

137 4; 139 2; 141 3; 142 1; 143 9;

144 5; 147 470; 148 67; 149 35; 151 2;

155 6; 156 1; 157 8; 158 2; 161 2;

164 4; 170 25; 171 1; 173 8; 174 5;

178 2; 184 2; 185 6; 190 1; 193 1;

195 1; 199 12; 200 6; 201 3; 205 3;

214 11; 215 13; 216 5; 220 1; 223 1;

230 7; 231 2; 236 2; 240 1; 245 2;

256 3; 257 7; 259 12; 260 7; 271 1;

273 7; 275 4; 277 1; 283 1; 286 1;

288 4; 290 4; 294 1; 296 2; 302 7;

304 1; 313 4; 315 1; 321 3; 330 3;

332 1; 341 2; 343 2; 344 5; 348 1;

354 3; 355 1; 365 3; 367 5; 372 4;

373 1; 374 3; 376 1; 377 3; 379 9;

381 3; 387 2; 389 1; 396 7; 399 3;

400 6; 401 8; 404 1; 407 3; 408 1;

410 8; 412 2; 416 3; 417 3; 418 2;

419 3; 421 5; 422 3; 423 3; 424 2;

425 4; 427 2; 429 8; 430 3; 432 3;

436 7; 443 1; 444 2; 445 1; 446 7;

447 3; 450 1; 452 6; 453 1; 457 1;

462 6; 463 1; 465 8; 467 4; 470 2;

471 5; 474 5; 475 3; 476 4; 477 2;

478 1; 479 2; 482 1; 487 4; 489 2;

490 1; 494 1; 497 2; 498 1; 499 2;

501 5; 502 4; 505 3; 507 1; 509 2;

512 3; 513 2; 514 6; 516 3; 517 2;

518 8; 519 4; 520 3; 522 5; 523 6;

525 4; 526 6; 528 5; 530 6; 533 8;

539 4; 541 1; 543 1; 545 6; 547 1;

548 4; 549 7; 551 2; 552 6; 559 3;

560 2; 561 1; 562 1; 563 1; 565 2;

566 1; 569 3; 570 2; 573 2; 575 1;

576 2; 577 3; 581 1; 583 1; 585 1;

586 1; 587 5; 588 5; 589 2; 590 7;

591 2; 592 1; 594 3; 595 2; 596 3;

597 4; 598 1;

Name: C12_POL_1813.26_433

Synon: Metabolite name: non-identified

Synon: Note: similar to pinitol (5TMS)

Synon: RI: 1813 iu

DB#: 7

Num Peaks: 188

70 2; 71 41; 72 14; 73 999; 74 88;

75 86; 76 4; 77 3; 81 12; 82 2;

83 4; 84 2; 85 6; 86 1; 87 3;

88 1; 89 87; 90 5; 91 4; 95 1;

97 1; 98 1; 99 5; 100 1; 101 9;

102 3; 103 139; 104 12; 105 8; 109 2;

111 4; 113 3; 114 1; 115 4; 116 11;

117 18; 118 2; 119 7; 120 1; 121 1;

125 1; 126 1; 127 3; 128 1; 129 84;

130 9; 131 27; 132 3; 133 131; 134 14;

135 7; 139 1; 141 1; 142 4; 143 15;

144 3; 145 5; 146 5; 147 197; 148 34;

149 21; 150 2; 151 1; 153 1; 155 3;

156 2; 157 8; 158 1; 159 71; 160 8;

161 6; 162 1; 163 21; 164 3; 165 1;

167 1; 169 2; 170 1; 171 1; 172 2;

173 20; 174 3; 175 3; 177 16; 178 2;

179 1; 183 1; 184 1; 185 2; 186 1;

187 1; 189 9; 190 5; 191 101; 192 15;

193 7; 194 1; 197 1; 198 1; 199 1;

201 2; 202 1; 203 4; 204 31; 205 23;

206 5; 207 45; 208 7; 209 3; 213 1;

215 2; 216 1; 217 143; 218 30; 219 12;

220 1; 221 8; 222 1; 223 1; 228 1;

230 4; 231 5; 232 1; 233 14; 234 2;

235 2; 243 5; 244 1; 245 4; 246 2;

247 39; 248 6; 249 2; 255 1; 259 2;

260 109; 261 24; 262 8; 263 3; 264 1;

265 13; 266 3; 267 1; 271 2; 277 2;

278 1; 285 2; 291 2; 292 1; 293 2;

304 2; 305 34; 306 9; 307 9; 308 2;

309 1; 317 3; 318 54; 319 16; 320 7;

321 1; 331 1; 335 2; 336 1; 342 1;

343 14; 344 5; 345 4; 346 1; 359 2;

361 2; 367 2; 368 1; 373 1; 374 8;

375 5; 376 2; 377 1; 417 1; 418 1;

432 5; 433 6; 434 3; 435 1; 448 1;

449 6; 450 3; 451 2;

Name: C13_POL_1571.41_179

Synon: Metabolite name: Tyrosol

Synon: Analyte name: M000852_A157014-101-xxx_NA_1,575.25_TRUE_VAR5_ALK_Ethanol, 2-(4-hydroxyphenyl)- (2TMS)

Synon: Analyte name: Tyrosol, 2TMS derivative

Synon: RI: 1571 iu

Synon: Formula: C14H26O2Si2

Synon: MW: 282 Exact Mass: 282.147133 CAS#: 321884-10-0

DB#: 39

Num Peaks: 403

73 901; 74 73; 75 100; 76 9; 77 46;

78 20; 79 4; 80 5; 81 8; 82 29;

83 3; 84 8; 86 5; 88 2; 89 36;

90 12; 91 43; 92 5; 93 4; 95 1;

97 7; 98 2; 102 2; 103 214; 104 21;

105 24; 106 1; 108 2; 109 7; 113 3;

114 4; 115 13; 117 32; 118 6; 119 6;

121 14; 122 11; 123 4; 126 42; 127 6;

128 6; 132 5; 135 17; 136 2; 137 2;

138 2; 140 1; 141 2; 142 4; 143 8;

144 2; 145 3; 146 7; 151 11; 152 3;

153 3; 154 4; 155 3; 156 28; 161 16;

162 5; 163 13; 164 8; 166 4; 167 3;

168 3; 169 7; 170 7; 171 3; 174 9;

175 7; 176 2; 177 21; 178 9; 179 999;

180 137; 181 39; 182 9; 184 4; 185 4;

186 5; 187 3; 188 6; 189 7; 191 5;

192 3; 193 81; 194 15; 195 6; 198 18;

199 3; 200 1; 201 4; 202 2; 208 1;

210 3; 211 2; 212 2; 213 2; 214 3;

216 1; 220 2; 224 2; 225 2; 227 1;

228 1; 229 4; 232 1; 235 5; 236 5;

237 2; 238 1; 239 1; 240 4; 242 1;

243 3; 244 2; 245 5; 250 2; 252 2;

253 6; 254 7; 255 1; 256 2; 257 3;

258 4; 259 2; 260 3; 261 4; 262 4;

263 4; 264 4; 265 4; 266 1; 267 52;

268 16; 269 9; 271 7; 272 3; 273 2;

274 5; 275 5; 276 3; 277 4; 278 1;

279 2; 280 5; 281 4; 282 70; 283 24;

284 7; 285 4; 286 2; 287 3; 288 5;

289 6; 290 1; 291 3; 293 5; 294 2;

295 4; 296 3; 297 6; 298 2; 299 2;

300 5; 301 3; 302 5; 303 4; 304 8;

305 5; 306 6; 307 2; 308 4; 309 2;

310 4; 312 3; 313 1; 314 3; 315 7;

316 1; 317 2; 318 3; 319 1; 320 1;

323 3; 327 3; 328 4; 329 5; 330 3;

331 2; 332 5; 333 3; 334 4; 335 2;

336 4; 337 2; 338 2; 339 2; 340 2;

341 2; 342 3; 343 3; 344 2; 346 3;

348 2; 352 4; 353 2; 354 3; 355 5;

356 2; 357 1; 359 6; 360 5; 361 6;

362 6; 363 6; 364 4; 369 3; 371 3;

372 1; 374 2; 375 2; 376 3; 377 3;

378 4; 379 4; 380 5; 381 4; 382 1;

383 2; 384 8; 385 3; 386 2; 387 3;

388 1; 389 5; 390 4; 391 2; 392 3;

393 5; 394 2; 395 6; 396 2; 398 1;

399 1; 400 2; 401 1; 402 3; 403 1;

404 3; 405 4; 406 2; 407 3; 408 2;

409 1; 411 3; 412 2; 413 4; 414 4;

415 5; 416 5; 417 2; 418 2; 419 6;

421 4; 422 2; 424 3; 425 5; 426 4;

427 4; 428 3; 430 2; 431 1; 433 1;

434 3; 435 2; 436 2; 437 1; 438 6;

439 6; 440 2; 441 3; 442 3; 443 3;

444 4; 445 3; 446 2; 447 2; 448 4;

449 1; 450 1; 451 4; 453 1; 454 2;

455 1; 457 5; 458 1; 459 2; 461 3;

462 1; 464 6; 465 5; 466 7; 467 4;

469 3; 470 2; 471 3; 472 5; 474 1;

475 2; 476 2; 477 6; 478 4; 479 2;

480 4; 481 4; 483 3; 484 1; 486 3;

487 1; 488 7; 489 2; 490 4; 491 1;

492 4; 493 3; 494 8; 495 3; 496 3;

499 1; 503 1; 504 4; 507 1; 508 1;

510 1; 511 1; 514 1; 515 2; 516 2;

517 5; 519 1; 520 2; 521 1; 522 1;

523 5; 525 3; 527 1; 528 1; 529 3;

531 1; 532 1; 533 2; 534 3; 535 1;

536 1; 538 2; 539 2; 540 3; 541 2;

542 4; 545 4; 546 1; 548 4; 549 2;

550 3; 552 4; 553 5; 554 3; 556 3;

557 1; 558 3; 559 2; 560 2; 561 2;

562 4; 563 5; 564 1; 565 1; 567 3;

570 2; 571 1; 572 1; 573 6; 574 3;

575 1; 577 3; 579 2; 580 3; 583 1;

584 2; 585 1; 586 2; 587 5; 588 2;

589 7; 590 1; 591 3; 592 1; 593 3;

594 2; 595 5; 596 2;

Name: C14_POL_2970.13_84

Synon: Metabolite name: non-identified

Synon: Note: also identified as M09

Synon: RI: 2970 iu

DB#: 21

Num Peaks: 221

70 4; 71 6; 72 10; 73 999; 74 82;

75 95; 76 6; 77 5; 79 2; 80 1;

81 21; 82 2; 83 5; 84 13; 85 13;

86 2; 87 5; 88 1; 89 11; 90 1;

91 1; 94 1; 95 2; 97 5; 98 1;

99 6; 100 4; 101 31; 102 5; 103 107;

104 10; 105 6; 107 1; 109 7; 110 1;

111 3; 112 1; 113 8; 114 2; 115 10;

116 13; 117 51; 118 5; 119 8; 120 1;

121 1; 125 1; 126 1; 127 5; 128 1;

129 149; 130 21; 131 32; 132 4; 133 32;

134 4; 135 3; 136 1; 139 2; 140 3;

141 13; 142 5; 143 28; 144 3; 145 12;

146 1; 147 147; 148 25; 149 22; 150 2;

151 2; 152 1; 153 2; 154 2; 155 12;

156 2; 157 19; 158 3; 159 4; 160 1;

161 4; 162 1; 163 3; 164 1; 167 2;

168 1; 169 33; 170 5; 171 8; 172 13;

173 21; 174 5; 175 4; 176 1; 177 4;

178 1; 179 1; 181 1; 182 1; 183 4;

184 1; 185 2; 186 4; 187 2; 188 1;

189 31; 190 7; 191 38; 192 6; 193 3;

195 1; 197 1; 199 6; 200 2; 201 2;

202 1; 203 11; 204 652; 205 131; 206 60;

207 10; 208 2; 214 1; 215 3; 216 2;

217 100; 218 31; 219 13; 220 3; 221 4;

222 1; 223 1; 227 1; 228 1; 229 3;

230 3; 231 9; 232 2; 233 3; 234 1;

239 1; 240 1; 241 1; 242 2; 243 18;

244 4; 245 5; 246 2; 247 3; 248 1;

255 1; 256 1; 257 3; 258 1; 259 2;

260 1; 261 1; 263 1; 265 1; 266 1;

270 1; 271 14; 272 4; 273 5; 274 1;

275 1; 282 1; 287 1; 289 2; 290 1;

291 3; 292 1; 293 1; 298 1; 300 1;

303 1; 304 1; 305 8; 306 3; 307 2;

314 1; 317 4; 318 2; 319 4; 320 1;

321 1; 331 3; 332 3; 333 3; 334 1;

345 1; 346 1; 347 1; 356 2; 357 1;

359 1; 360 2; 361 29; 362 11; 363 6;

364 2; 365 1; 388 2; 389 1; 435 1;

437 1; 445 2; 446 15; 447 7; 448 3;

449 1; 451 1; 462 1; 505 1; 506 3;

507 1; 508 1; 563 1; 564 4; 565 3;

566 1;

Name: C15_POL_3025.02_84

Synon: Metabolite name: non-identified

Synon: Note: also identified as M10

Synon: RI: 3025 iu

DB#: 5

Num Peaks: 191

70 4; 71 6; 72 30; 73 999; 74 85;

75 98; 76 6; 77 4; 79 1; 80 1;

81 21; 82 17; 83 6; 84 11; 85 10;

86 1; 87 3; 88 1; 89 9; 91 1;

94 1; 95 1; 97 6; 98 1; 99 6;

100 3; 101 29; 102 4; 103 109; 104 10;

105 5; 109 7; 111 3; 112 1; 113 7;

114 1; 115 8; 116 13; 117 53; 118 4;

119 8; 120 1; 125 1; 126 1; 127 5;

128 1; 129 155; 130 21; 131 26; 132 3;

133 32; 134 4; 135 3; 139 1; 140 4;

141 3; 142 4; 143 27; 144 3; 145 15;

146 2; 147 146; 148 24; 149 22; 150 2;

151 2; 153 2; 154 6; 155 19; 156 3;

157 20; 158 3; 159 4; 161 4; 163 3;

164 1; 167 1; 168 1; 169 32; 170 5;

171 8; 172 12; 173 10; 174 3; 175 4;

177 4; 178 1; 181 1; 183 4; 184 1;

185 1; 187 10; 188 2; 189 32; 190 7;

191 37; 192 5; 193 2; 194 1; 195 1;

197 1; 199 6; 200 5; 201 2; 202 1;

203 10; 204 654; 205 136; 206 62; 207 9;

208 2; 213 1; 214 1; 215 3; 216 1;

217 101; 218 28; 219 11; 220 2; 221 3;

222 1; 227 1; 228 1; 229 3; 230 3;

231 8; 232 2; 233 3; 241 1; 242 2;

243 16; 244 4; 245 4; 246 2; 247 3;

255 1; 256 1; 257 2; 258 1; 259 2;

261 1; 263 1; 271 14; 272 3; 273 4;

274 1; 275 1; 280 1; 282 1; 286 1;

287 1; 288 1; 289 2; 291 2; 292 1;

293 1; 304 1; 305 7; 306 2; 307 1;

314 1; 317 4; 318 2; 319 3; 320 1;

331 3; 332 3; 333 2; 345 1; 360 2;

361 29; 362 10; 363 6; 364 1; 365 1;

370 1; 388 1; 459 2; 460 13; 461 6;

462 3; 463 1; 505 1; 506 3; 507 1;

508 1; 577 1; 578 4; 579 2; 580 1;

581 1;

Name: C16_POL_2091.88_218

Synon: Metabolite name: myo-Inositol

Synon: Analyte name: M000060_A209002-101-xxx_NA_2,080.20_TRUE_VAR5_ALK_Inositol, myo- (6TMS)

Synon: Analyte name: myo-Inositol (6TMS)

Synon: RI: 2092 iu

Synon: Formula: C24H60O6Si6

Synon: MW: 612 Exact Mass: 612.30055 CAS#: 2582-79-8

DB#: 15

Num Peaks: 146

70 1; 71 1; 72 11; 73 999; 74 81;

75 58; 76 2; 77 2; 79 1; 81 8;

82 1; 83 4; 84 1; 85 3; 86 1;

87 3; 88 1; 89 1; 99 2; 101 6;

102 2; 103 80; 104 8; 105 4; 109 2;

111 3; 113 3; 115 3; 116 3; 117 7;

118 1; 119 3; 125 1; 126 1; 127 3;

128 1; 129 83; 130 9; 131 27; 132 3;

133 61; 134 8; 135 5; 136 1; 139 1;

140 1; 141 2; 142 1; 143 20; 144 2;

145 2; 146 1; 147 267; 148 43; 149 27;

150 2; 151 1; 153 1; 155 1; 156 1;

157 6; 158 1; 159 2; 161 6; 162 1;

163 1; 169 1; 173 1; 175 2; 176 1;

177 7; 178 1; 179 1; 181 1; 189 9;

190 6; 191 134; 192 27; 193 12; 194 1;

201 1; 203 4; 204 51; 205 14; 206 4;

207 6; 208 1; 209 1; 215 2; 216 2;

217 216; 218 47; 219 21; 220 2; 221 23;

222 4; 223 2; 229 1; 230 3; 231 2;

243 5; 244 1; 245 1; 264 1; 265 41;

266 8; 267 4; 268 1; 271 1; 291 10;

292 3; 293 4; 294 1; 303 1; 304 7;

305 138; 306 46; 307 24; 308 4; 309 1;

317 5; 318 73; 319 35; 320 13; 321 3;

322 1; 343 2; 344 1; 345 1; 366 1;

367 8; 368 3; 369 1; 392 1; 393 4;

394 1; 395 1; 419 1; 431 1; 432 11;

433 8; 434 4; 435 1; 507 2; 508 1;

509 1;

Name: M01_POL_1625.4_423

Synon: Seed: Linseed

Synon: Metabolite name: Tartaric acid

Synon: Analyte name: M000575_A164006-101-xxx_NA_1,628.94_TRUE_VAR5_ALK_Tartaric acid (4TMS)

Synon: Analyte name: Tartaric acid, 4TMS derivative

Synon: Formula: C16H38O6Si4

Synon: MW: 438 Exact Mass: 438.174545 CAS#: 18602-86-3

Synon: RI: 1625 iu

DB#: 79

Num Peaks: 122

70 3; 71 4; 72 16; 73 999; 74 81;

75 51; 76 2; 77 1; 83 1; 84 1;

85 3; 87 4; 88 1; 89 1; 99 5;

101 4; 102 50; 103 19; 104 4; 105 3;

113 1; 115 6; 116 2; 117 12; 118 1;

119 4; 120 1; 127 1; 129 1; 130 22;

131 19; 132 4; 133 33; 134 5; 135 3;

142 1; 143 41; 144 6; 145 3; 147 255;

148 38; 149 22; 150 3; 151 1; 157 1;

159 1; 161 2; 163 5; 164 1; 165 1;

171 12; 172 2; 173 2; 175 16; 176 3;

177 4; 178 1; 185 1; 189 89; 190 17;

191 17; 193 1; 204 6; 205 3; 206 1;

207 6; 208 1; 209 1; 215 1; 217 16;

219 74; 220 13; 221 29; 222 7; 223 3;

224 1; 231 4; 232 1; 233 1; 245 1;

259 1; 261 1; 263 9; 264 2; 265 1;

277 10; 278 4; 279 4; 280 1; 291 5;

292 119; 293 33; 294 16; 295 3; 296 1;

304 2; 305 27; 306 10; 307 5; 308 1;

321 6; 322 2; 323 1; 332 1; 333 17;

334 6; 335 3; 336 1; 351 5; 352 2;

353 1; 367 4; 368 2; 369 1; 395 1;

396 1; 422 4; 423 25; 424 10; 425 5;

426 1; 438 1;

Name: M02_POL_1837.1_171

Synon: Seed: Sesame

Synon: Metabolite name: non-identified

Synon: RI: 1837 iu

DB#: 76

Num Peaks: 206

70 2; 71 3; 72 17; 73 999; 74 86;

75 180; 76 11; 77 21; 78 3; 79 25;

80 3; 81 29; 82 6; 83 10; 84 1;

85 8; 87 4; 88 1; 89 4; 90 1;

91 20; 92 2; 93 8; 94 1; 95 6;

96 1; 97 11; 98 2; 99 8; 101 17;

102 2; 103 56; 104 5; 105 35; 106 5;

107 39; 108 3; 109 6; 110 2; 111 8;

112 2; 113 4; 114 2; 115 14; 116 6;

117 36; 118 3; 119 8; 120 1; 121 7;

122 1; 123 12; 124 2; 125 3; 126 1;

127 9; 128 1; 129 15; 130 14; 131 22;

132 6; 133 75; 134 7; 135 6; 136 1;

137 2; 138 1; 139 2; 140 1; 141 14;

142 5; 143 23; 144 2; 145 2; 147 224;

148 39; 149 40; 150 4; 151 7; 152 2;

153 3; 154 2; 155 31; 156 8; 157 43;

158 4; 159 3; 161 2; 162 1; 163 8;

164 1; 165 4; 166 1; 167 6; 168 8;

169 26; 170 4; 171 84; 172 9; 173 4;

175 1; 177 3; 178 1; 179 12; 180 15;

181 40; 182 8; 183 6; 184 1; 185 1;

189 2; 191 20; 192 2; 193 3; 194 17;

195 58; 196 11; 197 7; 198 1; 199 1;

201 1; 202 9; 203 2; 204 1; 205 3;

207 7; 208 1; 209 4; 210 1; 211 1;

213 1; 215 2; 216 1; 217 39; 218 6;

219 3; 221 8; 222 14; 223 39; 224 7;

225 3; 228 1; 229 3; 230 2; 231 103;

232 23; 233 7; 234 1; 239 1; 240 4;

241 2; 242 1; 243 2; 244 3; 245 3;

246 10; 247 9; 248 2; 249 1; 253 1;

255 2; 256 1; 257 4; 258 1; 259 2;

260 1; 261 22; 262 4; 263 2; 268 2;

269 4; 270 2; 271 4; 272 15; 273 5;

274 1; 283 1; 284 12; 285 27; 286 9;

287 3; 296 1; 297 11; 298 3; 299 5;

300 1; 301 1; 311 5; 312 102; 313 27;

314 8; 315 2; 319 1; 333 2; 334 1;

347 1; 359 1; 360 1; 361 1; 387 3;

388 1; 389 1; 401 1; 402 2; 403 1;

404 1;

Name: M03_POL_1946.26_306

Synon: Seed: Linseed

Synon: Metabolite name: non-identified

Synon: Note: similar to gluconic acid (6TMS)

Synon: RI: 1946 iu

DB#: 88

Num Peaks: 165

72 7; 73 999; 74 81; 75 51; 76 2;

81 2; 83 10; 84 1; 85 2; 87 4;

88 2; 89 14; 90 1; 97 4; 99 3;

101 13; 102 20; 103 104; 104 11; 105 4;

107 1; 111 1; 113 3; 115 4; 116 3;

117 64; 118 6; 119 5; 127 2; 129 70;

130 22; 131 29; 132 4; 133 48; 134 6;

135 4; 141 2; 142 1; 143 40; 144 4;

145 4; 146 1; 147 334; 148 53; 149 36;

150 3; 151 1; 153 2; 155 1; 157 45;

158 5; 159 3; 161 2; 163 3; 169 7;

170 1; 171 9; 172 1; 173 2; 175 5;

176 1; 177 2; 185 1; 187 1; 189 43;

190 11; 191 26; 192 4; 193 1; 197 3;

201 2; 202 1; 203 4; 204 36; 205 99;

206 23; 207 15; 208 2; 209 1; 215 2;

216 1; 217 77; 218 18; 219 16; 220 5;

221 19; 222 4; 223 2; 227 1; 229 16;

230 3; 231 5; 232 1; 233 1; 241 1;

243 2; 244 1; 245 7; 246 1; 247 1;

257 1; 259 2; 260 1; 261 1; 263 1;

265 1; 269 2; 271 1; 277 9; 278 3;

279 2; 290 1; 291 11; 292 86; 293 29;

294 13; 295 2; 304 2; 305 35; 306 11;

307 10; 308 2; 309 1; 315 1; 316 1;

317 1; 318 4; 319 62; 320 20; 321 10;

322 2; 323 1; 330 1; 331 8; 332 8;

333 83; 334 30; 335 13; 336 2; 343 1;

344 1; 345 3; 346 1; 358 1; 359 12;

360 3; 361 1; 379 1; 389 1; 393 1;

394 1; 405 1; 406 1; 407 1; 421 1;

422 1; 423 5; 424 2; 425 1; 432 1;

433 8; 434 3; 435 5; 436 2; 437 1;

Name: M04_POL_2003.15_333

Synon: Seed: Linseed

Synon: Metabolite name: Saccharic acid

Synon: Analyte name: M000093_A201001-101-xxx_NA_2,000.79_TRUE_VAR5_ALK_Saccharic acid (6TMS)

Synon: Analyte name: Galactaric acid, 6TMS derivative

Synon: Formula: C24H58O8Si6

Synon: MW: 642 Exact Mass: 642.274727 CAS#: 38165-96-7

Synon: RI: 2003 iu

DB#: 68

Num Peaks: 166

70 2; 71 3; 72 13; 73 999; 74 82;

75 57; 76 3; 77 2; 81 1; 83 5;

84 2; 85 5; 86 1; 87 5; 88 1;

89 2; 95 2; 97 1; 98 1; 99 6;

100 1; 101 8; 102 23; 103 28; 104 4;

105 3; 111 2; 113 3; 114 1; 115 4;

116 3; 117 9; 118 1; 119 4; 125 1;

127 4; 128 1; 129 13; 130 9; 131 16;

132 4; 133 27; 134 4; 135 3; 141 2;

142 1; 143 53; 144 7; 145 5; 146 1;

147 201; 148 30; 149 18; 150 2; 151 1;

155 1; 156 1; 157 3; 159 2; 161 3;

163 3; 169 2; 171 17; 172 3; 173 3;

175 4; 176 1; 177 2; 183 2; 185 1;

187 1; 189 36; 190 8; 191 15; 192 3;

193 2; 201 2; 203 2; 204 12; 205 5;

206 2; 207 6; 208 1; 209 1; 211 1;

215 2; 217 26; 218 5; 219 12; 220 4;

221 18; 222 5; 223 3; 224 1; 229 4;

230 1; 231 4; 232 1; 233 1; 243 1;

245 6; 246 2; 247 1; 249 1; 257 3;

258 1; 259 1; 261 1; 263 1; 265 1;

277 12; 278 4; 279 3; 280 1; 291 4;

292 43; 293 13; 294 6; 295 1; 303 1;

304 1; 305 16; 306 5; 307 3; 308 1;

317 1; 318 1; 319 1; 320 1; 321 2;

322 1; 331 1; 332 1; 333 99; 334 25;

335 9; 336 2; 345 3; 346 1; 347 1;

373 9; 374 4; 375 2; 379 3; 380 1;

381 1; 393 3; 394 1; 395 1; 419 5;

420 2; 421 1; 422 1; 423 8; 424 4;

425 2; 435 4; 436 2; 437 1; 447 4;

448 2; 449 1; 493 1; 509 2; 510 1;

537 1;

Name: M05_POL_2045.05_231

Synon: Seed: Sesame

Synon: Metabolite name: non-identified

Synon: RI: 2045 iu

DB#: 145

Num Peaks: 289

70 3; 71 9; 72 21; 73 999; 74 77;

75 317; 76 24; 77 27; 78 3; 79 17;

80 2; 81 31; 82 5; 83 24; 84 5;

85 11; 86 2; 87 5; 88 2; 89 79;

90 7; 91 19; 92 2; 93 10; 94 1;

95 33; 96 3; 97 17; 98 9; 99 11;

101 13; 102 2; 103 53; 104 7; 105 53;

106 5; 107 9; 108 2; 109 17; 110 2;

111 15; 112 4; 113 4; 114 1; 115 17;

116 17; 117 197; 118 22; 119 26; 120 4;

121 17; 122 1; 123 15; 124 2; 125 5;

126 2; 127 6; 128 2; 129 101; 130 21;

131 67; 132 87; 133 82; 134 13; 135 14;

136 2; 137 5; 138 1; 139 15; 140 2;

141 13; 142 4; 143 57; 144 9; 145 57;

146 7; 147 180; 148 34; 149 47; 150 5;

151 7; 152 2; 153 10; 154 3; 155 7;

156 5; 157 24; 158 3; 159 43; 160 9;

161 24; 162 3; 163 30; 164 4; 165 10;

166 2; 167 17; 168 8; 169 29; 170 5;

171 10; 172 2; 173 4; 174 1; 175 3;

176 16; 177 10; 178 2; 179 9; 180 3;

181 8; 182 2; 183 11; 184 2; 185 9;

186 2; 187 5; 188 1; 189 2; 190 3;

191 56; 192 7; 193 14; 194 4; 195 12;

196 5; 197 13; 198 2; 199 9; 200 2;

201 13; 202 7; 203 2; 204 1; 205 8;

206 2; 207 15; 208 4; 209 9; 210 1;

211 2; 212 1; 213 7; 214 2; 215 4;

216 1; 217 57; 218 11; 219 6; 220 2;

221 21; 222 4; 223 7; 224 2; 225 4;

226 1; 227 5; 228 1; 229 19; 230 4;

231 90; 232 24; 233 10; 234 2; 235 1;

236 2; 237 8; 238 3; 239 14; 240 11;

241 12; 242 3; 243 6; 244 3; 245 5;

246 8; 247 17; 248 6; 249 25; 250 4;

251 5; 252 5; 253 13; 254 3; 255 3;

256 1; 257 9; 258 3; 259 2; 260 1;

261 1; 265 3; 266 1; 267 3; 268 1;

269 6; 270 2; 271 4; 272 1; 273 2;

274 1; 279 1; 280 12; 281 15; 282 3;

283 5; 284 12; 285 112; 286 36; 287 15;

288 3; 289 1; 295 3; 296 1; 297 2;

298 2; 299 2; 300 1; 301 1; 303 1;

305 3; 306 1; 307 1; 308 1; 309 1;

310 3; 311 4; 312 5; 313 52; 314 13;

315 4; 316 1; 317 1; 318 1; 319 3;

320 1; 321 1; 323 3; 324 1; 325 2;

326 2; 327 3; 328 4; 329 2; 330 4;

331 2; 332 1; 333 4; 334 2; 335 1;

337 1; 338 6; 339 5; 340 2; 341 1;

342 3; 343 6; 344 2; 345 1; 346 1;

354 1; 355 4; 356 1; 357 2; 358 1;

359 1; 369 2; 370 22; 371 5; 372 2;

373 3; 374 1; 375 3; 376 1; 386 1;

387 1; 391 1; 403 1; 413 1; 430 1;

444 2; 445 13; 446 5; 447 2; 448 1;

460 1; 534 1; 535 3; 536 1;

Name: M06_POL_2051.2_267

Synon: Seed: Chia

Synon: Metabolite name: Catechollactic acid

Synon: Analyte name: Trimethylsilyl catechollactate tris(trimethylsilyl) ether

Synon: Formula: C21H42O5Si4

Synon: MW: 486 Exact Mass: 486.21093 CAS#: 68595-72-2

Synon: RI: 2051 iu

DB#: 71

Num Peaks: 118

70 2; 71 2; 72 9; 73 999; 74 87;

75 67; 76 3; 77 8; 78 2; 82 4;

83 1; 84 1; 87 2; 88 2; 89 3;

91 5; 93 1; 95 1; 97 1; 99 1;

101 1; 102 4; 103 6; 104 3; 105 7;

109 1; 115 6; 116 2; 117 9; 118 1;

119 5; 120 1; 121 1; 129 3; 130 1;

131 16; 132 2; 133 23; 134 2; 135 4;

136 1; 137 1; 145 1; 147 128; 148 17;

149 25; 150 3; 151 2; 159 1; 160 1;

161 3; 162 1; 163 4; 164 1; 165 1;

175 2; 177 4; 178 1; 179 168; 180 20;

181 6; 189 1; 191 18; 192 4; 193 5;

194 1; 195 1; 204 1; 205 3; 207 21;

208 3; 209 2; 219 24; 220 3; 221 3;

223 3; 224 1; 233 1; 235 1; 237 1;

249 3; 250 1; 251 2; 263 1; 265 8;

266 7; 267 299; 268 79; 269 25; 270 3;

279 2; 280 1; 281 4; 282 1; 292 4;

293 4; 294 1; 307 1; 309 1; 353 1;

355 1; 368 1; 369 1; 381 4; 382 1;

395 5; 396 38; 397 12; 398 5; 399 1;

443 1; 444 1; 471 2; 472 1; 485 1;

486 5; 487 2; 488 1;

Name: M07_POL_2209.11_109

Synon: Seed: Sesame

Synon: Metabolite name: non-identified

Synon: RI: 2209 iu

DB#: 128

Num Peaks: 355

72 14; 73 999; 74 80; 75 88; 76 5;

77 4; 79 3; 80 1; 81 11; 82 1;

83 14; 87 3; 88 1; 89 59; 90 5;

91 11; 92 2; 93 4; 95 18; 96 1;

97 23; 98 2; 99 5; 101 7; 102 2;

103 62; 104 6; 105 11; 106 1; 107 4;

108 2; 109 21; 110 1; 111 7; 112 1;

113 4; 114 2; 115 7; 116 4; 117 29;

118 3; 119 17; 120 2; 121 6; 123 9;

124 1; 125 4; 126 1; 127 4; 128 1;

129 25; 130 7; 131 25; 132 3; 133 56;

134 8; 135 12; 136 2; 137 6; 138 1;

139 9; 140 1; 141 20; 142 4; 143 35;

144 4; 145 6; 146 2; 147 293; 148 47;

149 41; 150 5; 151 10; 152 1; 153 5;

154 2; 155 6; 156 11; 157 31; 158 4;

159 44; 160 15; 161 9; 162 1; 163 26;

164 4; 165 6; 166 2; 167 13; 168 3;

169 37; 170 8; 171 5; 172 1; 173 3;

175 5; 176 2; 177 5; 178 1; 179 6;

180 2; 181 7; 182 3; 183 14; 184 3;

185 5; 186 1; 187 3; 188 1; 189 5;

190 1; 191 27; 192 5; 193 20; 194 4;

195 7; 196 3; 197 13; 198 2; 199 4;

200 2; 201 3; 202 11; 203 8; 204 16;

205 32; 206 7; 207 15; 208 3; 209 8;

210 2; 211 5; 212 5; 213 13; 214 2;

215 5; 216 1; 217 20; 218 285; 219 64;

220 29; 221 21; 222 4; 223 4; 224 1;

225 4; 226 1; 227 5; 228 1; 229 3;

230 4; 231 57; 232 12; 233 6; 234 1;

235 4; 236 1; 237 5; 238 1; 239 5;

240 38; 241 13; 242 3; 243 9; 244 3;

245 19; 246 113; 247 169; 248 46; 249 18;

250 4; 251 12; 252 3; 253 5; 254 2;

255 6; 256 2; 257 20; 258 14; 259 14;

260 4; 261 1; 263 1; 264 1; 265 5;

266 1; 267 4; 268 1; 269 3; 270 3;

271 7; 272 2; 273 5; 274 2; 275 1;

277 1; 278 6; 279 19; 280 4; 281 7;

282 2; 283 6; 284 3; 285 3; 286 5;

287 4; 288 2; 289 3; 290 1; 291 1;

293 1; 294 1; 295 2; 296 1; 297 3;

298 5; 299 5; 300 2; 301 3; 302 3;

303 2; 304 1; 305 2; 306 1; 307 2;

308 2; 309 11; 310 4; 311 4; 312 1;

313 2; 314 1; 315 4; 316 1; 317 2;

318 2; 319 15; 320 4; 321 2; 322 1;

323 2; 324 2; 325 6; 326 3; 327 4;

328 2; 329 4; 330 40; 331 15; 332 5;

333 2; 334 2; 335 1; 336 3; 337 7;

338 2; 339 2; 340 4; 341 5; 342 2;

343 2; 344 1; 345 1; 346 1; 347 1;

348 1; 352 1; 353 5; 354 2; 355 5;

356 2; 357 2; 358 1; 359 1; 360 1;

362 1; 363 2; 364 1; 367 3; 368 28;

369 23; 370 7; 371 3; 372 3; 373 16;

374 6; 375 3; 376 1; 377 1; 383 1;

384 1; 385 1; 386 1; 387 1; 388 1;

389 1; 390 1; 391 1; 392 2; 393 1;

398 1; 399 2; 400 1; 401 1; 402 1;

403 2; 404 2; 405 2; 406 1; 411 1;

412 1; 413 1; 414 1; 415 1; 416 1;

417 1; 418 1; 419 2; 420 8; 421 3;

422 1; 423 1; 426 1; 427 2; 428 1;

429 1; 430 1; 431 2; 432 1; 433 1;

442 1; 443 7; 444 3; 445 2; 446 1;

447 1; 457 2; 458 9; 459 4; 460 2;

461 1; 473 1; 501 1; 503 1; 504 1;

505 1; 506 1; 507 1; 532 1; 533 2;

534 1; 535 1; 547 1; 548 1; 549 1;

Name: M08_POL_2409.48_204

Synon: Seed: Sesame

Synon: Metabolite name: non-identified

Synon: RI: 2409 iu

DB#: 132

Num Peaks: 155

70 2; 71 16; 72 15; 73 999; 74 89;

75 147; 76 8; 77 6; 81 8; 82 2;

83 5; 84 1; 85 5; 86 1; 87 4;

88 2; 89 8; 90 1; 91 2; 94 1;

95 1; 97 2; 98 1; 99 10; 101 19;

102 3; 103 84; 104 6; 105 3; 109 3;

111 3; 113 6; 114 1; 115 7; 116 10;

117 42; 118 3; 119 5; 125 1; 127 3;

128 1; 129 138; 130 15; 131 25; 132 3;

133 39; 134 4; 135 3; 139 1; 141 2;

142 1; 143 38; 144 28; 145 19; 146 3;

147 199; 148 33; 149 31; 150 3; 151 2;

153 2; 154 1; 155 7; 156 1; 157 12;

158 2; 159 2; 160 1; 161 2; 163 12;

164 2; 169 38; 170 4; 171 5; 172 1;

173 4; 175 2; 177 2; 183 2; 185 1;

187 1; 189 22; 190 5; 191 36; 192 5;

193 3; 197 1; 199 2; 201 1; 202 1;

203 8; 204 423; 205 99; 206 38; 207 6;

208 1; 215 1; 216 1; 217 79; 218 21;

219 8; 220 1; 221 5; 222 1; 223 1;

227 1; 229 3; 230 2; 231 10; 232 3;

233 6; 234 2; 235 1; 241 1; 242 1;

243 13; 244 3; 245 5; 246 1; 247 2;

257 1; 258 1; 259 2; 260 1; 261 17;

262 3; 263 2; 265 1; 271 11; 272 2;

273 2; 291 3; 292 1; 293 1; 305 4;

306 5; 307 2; 308 1; 319 2; 321 1;

331 2; 332 1; 333 1; 334 1; 335 2;

345 1; 360 5; 361 61; 362 16; 363 7;

364 1; 379 3; 380 1; 381 1; 451 1;

Name: M09_POL_2978.07_360

Synon: Seed: Linseed

Synon: Metabolite name: non-identified

Synon: Note: also identified as C14

Synon: RI: 2978 iu

DB#: 104

Num Peaks: 253

70 4; 71 6; 72 10; 73 999; 74 77;

75 67; 76 4; 77 3; 79 1; 81 17;

82 2; 83 5; 84 7; 85 13; 86 2;

87 4; 88 2; 89 9; 90 1; 91 2;

94 1; 95 1; 97 5; 98 1; 99 5;

100 4; 101 23; 102 4; 103 104; 104 10;

105 5; 109 5; 110 1; 111 3; 112 1;

113 7; 114 2; 115 8; 116 9; 117 37;

118 4; 119 6; 120 1; 125 1; 126 1;

127 4; 128 1; 129 119; 130 15; 131 22;

132 4; 133 16; 134 3; 135 2; 139 1;

140 4; 141 15; 142 6; 143 18; 144 3;

145 11; 146 2; 147 109; 148 16; 149 12;

150 2; 151 1; 153 1; 154 1; 155 7;

156 2; 157 12; 158 3; 159 3; 160 1;

161 4; 162 1; 163 3; 164 1; 167 1;

168 1; 169 18; 170 4; 171 6; 172 9;

173 15; 174 4; 175 3; 176 1; 177 3;

181 1; 183 2; 184 1; 185 1; 186 4;

187 2; 188 1; 189 12; 190 4; 191 19;

192 4; 193 2; 195 1; 197 1; 198 1;

199 6; 200 2; 201 2; 202 1; 203 7;

204 500; 205 83; 206 30; 207 4; 208 1;

214 1; 215 4; 216 1; 217 95; 218 25;

219 12; 220 2; 221 4; 222 1; 223 1;

226 1; 227 1; 228 1; 229 5; 230 4;

231 11; 232 3; 233 5; 234 1; 235 1;

238 1; 239 1; 240 1; 241 2; 242 5;

243 22; 244 7; 245 8; 246 3; 247 6;

248 1; 249 1; 255 1; 256 1; 257 5;

258 1; 259 4; 260 1; 261 1; 263 1;

265 1; 266 3; 267 1; 268 1; 269 1;

270 1; 271 19; 272 8; 273 8; 274 2;

275 1; 277 1; 278 1; 279 1; 282 1;

285 1; 286 1; 287 1; 288 1; 289 4;

290 1; 291 5; 292 2; 293 1; 298 2;

299 1; 300 1; 301 1; 302 1; 303 1;

304 2; 305 14; 306 5; 307 3; 308 1;

313 1; 314 2; 315 1; 316 1; 317 9;

318 4; 319 7; 320 2; 321 1; 329 1;

330 1; 331 7; 332 7; 333 4; 334 1;

335 1; 345 3; 346 1; 347 1; 356 4;

357 1; 358 1; 359 1; 360 3; 361 39;

362 13; 363 10; 364 3; 365 1; 372 1;

379 1; 388 4; 389 1; 390 1; 393 1;

403 1; 405 1; 406 1; 407 2; 408 1;

430 1; 431 1; 432 1; 433 1; 435 1;

445 4; 446 33; 447 13; 448 6; 449 1;

462 2; 463 1; 505 1; 506 8; 507 4;

508 3; 509 1; 562 1; 563 4; 564 17;

565 8; 566 4; 567 1;

Name: M10_POL_3032.18_172

Synon: Seed: Linseed

Synon: Metabolite name: non-identified

Synon: Note: also identified as C15

Synon: RI: 3032 iu

DB#: 107

Num Peaks: 236

70 3; 71 6; 72 19; 73 999; 74 81;

75 69; 76 4; 77 3; 79 1; 80 1;

81 17; 82 28; 83 6; 84 6; 85 11;

86 1; 87 4; 88 1; 89 9; 90 1;

91 2; 94 1; 95 1; 97 6; 98 1;

99 5; 100 4; 101 22; 102 4; 103 109;

104 11; 105 5; 109 7; 110 1; 111 3;

112 1; 113 8; 114 2; 115 6; 116 11;

117 40; 118 5; 119 8; 120 1; 125 1;

126 1; 127 4; 128 2; 129 132; 130 17;

131 18; 132 3; 133 17; 134 3; 135 2;

136 1; 139 1; 140 6; 141 3; 142 4;

143 16; 144 3; 145 15; 146 2; 147 112;

148 17; 149 13; 150 2; 151 1; 153 2;

154 8; 155 16; 156 3; 157 14; 158 3;

159 3; 160 1; 161 4; 162 1; 163 3;

167 1; 169 18; 170 4; 171 7; 172 10;

173 9; 174 3; 175 3; 176 1; 177 3;

178 1; 181 1; 183 3; 184 1; 185 1;

187 10; 188 2; 189 13; 190 5; 191 20;

192 4; 193 2; 195 1; 197 1; 198 1;

199 5; 200 5; 201 2; 202 1; 203 7;

204 477; 205 89; 206 36; 207 5; 208 1;

213 1; 214 1; 215 3; 216 1; 217 83;

218 22; 219 11; 220 2; 221 4; 222 1;

223 1; 226 1; 227 1; 228 1; 229 4;

230 4; 231 10; 232 2; 233 4; 234 1;

235 1; 241 1; 242 2; 243 16; 244 6;

245 6; 246 2; 247 5; 248 1; 254 1;

255 1; 256 2; 257 3; 258 1; 259 3;

260 1; 261 1; 263 1; 265 1; 270 1;

271 16; 272 5; 273 6; 274 1; 275 1;

279 1; 280 1; 282 1; 286 1; 287 1;

288 1; 289 3; 290 1; 291 4; 292 1;

293 1; 298 1; 300 1; 301 1; 303 1;

304 2; 305 13; 306 4; 307 2; 308 1;

314 1; 315 1; 316 1; 317 9; 318 3;

319 6; 320 1; 321 1; 328 1; 330 1;

331 6; 332 5; 333 3; 334 1; 335 1;

344 1; 345 2; 346 1; 347 1; 359 1;

360 4; 361 32; 362 12; 363 9; 364 2;

365 1; 370 3; 371 1; 372 1; 379 1;

388 3; 389 1; 390 1; 405 1; 406 1;

407 2; 408 1; 435 1; 444 1; 459 5;

460 26; 461 12; 462 7; 463 2; 464 1;

505 2; 506 8; 507 3; 508 2; 509 1;

576 1; 577 4; 578 13; 579 7; 580 3;

581 1;

Name: M11_POL_3145.68_205

Synon: Seed: Linseed

Synon: Metabolite name: non-identified

Synon: RI: 3146 iu

DB#: 109

Num Peaks: 181

70 5; 71 3; 72 10; 73 999; 74 87;

75 144; 76 8; 77 7; 79 8; 80 1;

81 16; 82 1; 83 3; 84 3; 85 7;

86 1; 87 3; 88 2; 89 9; 90 1;

91 17; 92 1; 93 3; 94 1; 97 3;

99 4; 100 2; 101 21; 102 4; 103 97;

104 9; 105 11; 106 2; 107 2; 109 4;

111 5; 112 1; 113 6; 114 1; 115 8;

116 16; 117 50; 118 6; 119 8; 120 1;

121 1; 125 1; 126 1; 127 3; 128 3;

129 129; 130 76; 131 28; 132 10; 133 50;

134 4; 135 3; 137 1; 139 2; 141 4;

142 17; 143 24; 144 4; 145 6; 146 5;

147 161; 148 20; 149 17; 150 1; 151 1;

153 3; 155 13; 156 2; 157 14; 158 21;

159 18; 160 3; 161 4; 162 1; 163 2;

164 1; 168 1; 169 35; 170 4; 171 4;

173 4; 174 2; 175 7; 176 1; 177 2;

179 1; 182 4; 183 3; 186 1; 187 1;

189 21; 190 3; 191 26; 192 3; 195 1;

196 1; 199 3; 200 1; 201 1; 203 4;

204 391; 205 67; 206 22; 207 5; 214 1;

215 1; 216 1; 217 88; 218 16; 219 8;

221 2; 223 1; 229 2; 230 2; 231 6;

232 8; 233 5; 238 1; 242 1; 243 11;

244 2; 245 1; 246 1; 248 1; 249 1;

255 1; 257 1; 259 3; 260 1; 264 1;

265 4; 266 1; 267 1; 270 2; 271 12;

272 4; 273 2; 281 1; 285 1; 286 1;

287 1; 289 2; 291 1; 305 4; 308 1;

310 1; 317 2; 318 1; 319 3; 320 1;

321 1; 331 1; 332 2; 356 1; 361 27;

362 8; 363 2; 364 2; 370 1; 391 1;

393 1; 414 1; 422 1; 458 1; 460 1;

466 1; 467 1; 489 1; 523 1; 538 1;

539 2; 540 1; 548 1; 586 1; 591 1;

593 1;

Name: M12_POL_3210.66_179

Synon: Seed: Chia

Synon: Metabolite name: Rosmarinic acid, cis-

Synon: Analyte name: M001162_A340009-101-xxx_NA_3,400.96_TRUE_VAR5_ALK_Rosmarinic acid (5TMS)

Synon: Analyte name: Rosmarinic acid, 5O-TMS

Synon: Formula: C33H56O8Si5

Synon: MW: 720 Exact Mass: 720.28215

Synon: RI: 3211 iu

DB#: 69

Num Peaks: 159

70 1; 72 8; 73 999; 74 90; 75 80;

76 5; 77 8; 78 1; 82 2; 83 3;

86 1; 87 2; 88 9; 89 10; 90 1;

91 3; 95 1; 99 1; 101 1; 102 5;

103 4; 104 2; 105 4; 107 1; 109 1;

114 1; 115 10; 116 3; 117 13; 118 1;

119 3; 121 1; 128 2; 129 2; 130 1;

131 8; 132 1; 133 12; 134 1; 135 9;

136 1; 137 3; 139 1; 141 2; 142 1;

143 2; 145 4; 147 16; 148 2; 149 8;

150 1; 151 2; 156 1; 157 1; 159 1;

160 3; 161 5; 162 2; 163 9; 164 2;

165 1; 166 1; 169 1; 171 1; 173 1;

174 1; 175 5; 176 1; 177 4; 178 1;

179 109; 180 13; 181 4; 182 1; 185 1;

186 1; 187 1; 189 2; 190 3; 191 72;

192 15; 193 8; 194 1; 195 1; 201 1;

203 4; 207 3; 208 1; 209 1; 210 1;

216 1; 217 2; 218 2; 219 170; 220 29;

221 8; 222 1; 223 2; 231 1; 232 1;

233 4; 234 1; 235 2; 236 3; 237 1;

247 1; 248 1; 249 13; 250 3; 251 2;

252 1; 261 1; 263 1; 264 1; 265 5;

266 2; 267 24; 268 5; 269 2; 277 2;

278 1; 279 6; 280 4; 281 2; 282 1;

289 1; 290 1; 291 5; 292 6; 293 12;

294 3; 295 1; 296 1; 297 1; 305 2;

306 2; 307 27; 308 9; 309 7; 310 1;

323 3; 324 56; 325 12; 326 4; 327 1;

337 1; 360 1; 361 1; 380 1; 381 9;

382 5; 383 3; 394 1; 395 19; 396 144;

397 58; 398 28; 399 6; 400 2;

Name: M13_POL_3399.31_396

Synon: Seed: Chia

Synon: Metabolite name: Rosmarinic acid, trans-

Synon: Analyte name: M001162_A340009-101-xxx_NA_3,400.96_TRUE_VAR5_ALK_Rosmarinic acid (5TMS)

Synon: Analyte name: Rosmarinic acid, 5O-TMS

Synon: Formula: C33H56O8Si5

Synon: MW: 720 Exact Mass: 720.28215

Synon: RI: 3399 iu

DB#: 70

Num Peaks: 170

70 1; 71 1; 72 9; 73 999; 74 83;

75 74; 76 5; 77 8; 78 1; 79 1;

82 2; 83 4; 84 1; 85 1; 86 1;

87 2; 88 12; 89 14; 90 1; 91 4;

93 1; 95 1; 99 1; 101 1; 102 6;

103 7; 104 2; 105 6; 106 1; 107 1;

109 2; 110 1; 115 14; 116 4; 117 20;

118 2; 119 5; 120 1; 121 1; 123 1;

127 1; 128 2; 129 6; 130 1; 131 11;

132 2; 133 16; 134 2; 135 12; 136 2;

137 3; 139 1; 141 2; 142 1; 143 2;

144 1; 145 5; 146 1; 147 25; 148 5;

149 11; 150 1; 151 1; 157 1; 159 1;

160 4; 161 7; 162 2; 163 11; 164 2;

165 1; 166 1; 173 1; 174 1; 175 7;

176 1; 177 5; 178 1; 179 100; 180 17;

181 5; 182 1; 185 1; 187 1; 188 1;

189 3; 190 4; 191 79; 192 21; 193 10;

194 1; 195 1; 201 1; 203 6; 204 2;

205 4; 206 1; 207 4; 208 1; 209 2;

216 1; 217 4; 218 3; 219 201; 220 39;

221 13; 222 1; 223 2; 231 1; 232 1;

233 6; 234 1; 235 2; 236 3; 237 2;

247 1; 248 1; 249 20; 250 4; 251 2;

252 1; 261 1; 263 2; 264 1; 265 7;

266 2; 267 29; 268 6; 269 2; 277 2;

278 1; 279 9; 280 7; 281 3; 282 1;

289 1; 290 1; 291 5; 292 8; 293 19;

294 5; 295 2; 296 1; 305 3; 306 3;

307 44; 308 16; 309 14; 310 3; 311 1;

323 3; 324 47; 325 13; 326 4; 327 1;

338 1; 339 1; 360 1; 380 2; 381 16;

382 7; 383 3; 384 1; 394 1; 395 25;

396 195; 397 73; 398 39; 399 9; 400 2;

Name: M14_SOL_1392.44_210

Synon: Seed: Sesame

Synon: Metabolite name: Sesamol

Synon: Analyte name: Sesamol, TMS derivative

Synon: Formula: C10H14O3Si

Synon: MW: 210 Exact Mass: 210.07122 CAS#: 17903-26-3

Synon: RI: 1392 iu

DB#: 159

Num Peaks: 206

70 91; 76 25; 77 211; 78 6; 79 190;

80 12; 81 1; 82 15; 83 185; 90 1;

93 1; 94 14; 97 242; 99 2; 101 1;

106 4; 107 29; 109 66; 110 1; 111 13;

113 2; 116 6; 117 2; 121 67; 122 36;

123 1; 124 1; 136 1; 137 654; 138 93;

139 6; 147 1; 151 7; 152 2; 155 19;

160 12; 165 993; 166 93; 167 148; 168 5;

169 77; 171 16; 172 3; 179 1; 182 40;

183 23; 189 1; 195 753; 196 77; 197 8;

204 7; 210 999; 211 166; 212 14; 217 7;

218 5; 219 2; 220 1; 234 3; 236 21;

237 23; 239 14; 240 12; 249 14; 250 2;

251 3; 252 10; 263 11; 264 4; 265 13;

269 2; 275 16; 276 4; 279 3; 285 2;

286 24; 289 4; 290 16; 293 2; 298 5;

300 4; 305 21; 306 6; 307 2; 308 6;

309 4; 311 2; 313 1; 314 1; 317 1;

319 2; 322 2; 326 5; 329 10; 331 1;

337 1; 340 1; 341 2; 347 9; 348 1;

353 3; 357 1; 358 9; 362 5; 364 7;

366 3; 367 5; 369 2; 370 2; 371 8;

372 1; 373 19; 382 3; 384 1; 390 7;

391 4; 393 1; 395 3; 396 10; 398 1;

399 1; 402 1; 403 3; 407 13; 409 2;

413 7; 417 1; 418 5; 421 1; 425 2;

429 3; 430 1; 432 5; 434 1; 435 2;

436 3; 440 6; 442 3; 443 4; 448 2;

450 2; 454 1; 456 1; 457 1; 459 7;

460 1; 465 2; 470 3; 471 1; 472 7;

473 1; 474 2; 475 7; 477 1; 478 1;

481 4; 483 2; 484 1; 485 1; 490 1;

491 1; 492 1; 494 8; 497 8; 498 5;

501 2; 506 2; 508 3; 511 1; 512 1;

514 3; 516 2; 520 6; 521 2; 525 3;

527 3; 531 3; 532 1; 533 2; 534 1;

538 4; 539 1; 541 6; 545 1; 548 1;

549 3; 552 1; 557 5; 558 7; 559 1;

561 2; 563 3; 564 1; 570 2; 571 3;

572 1; 574 1; 575 5; 580 1; 585 6;

586 2; 589 1; 596 7; 598 1; 599 11;

600 2;

Name: M15_SOL_2614.79_335

Synon: Seed: Chia

Synon: Metabolite name: non-identified

Synon: RI: 2615 iu

DB#: 147

Num Peaks: 268

70 4; 71 6; 72 14; 73 999; 74 86;

75 75; 76 5; 77 3; 79 1; 80 2;

81 1; 82 8; 83 4; 84 2; 85 5;

86 1; 87 3; 88 3; 89 34; 90 3;

91 2; 94 1; 95 2; 96 1; 97 1;

98 2; 99 4; 100 27; 101 15; 102 4;

103 111; 104 12; 105 9; 106 1; 107 1;

110 1; 111 3; 112 3; 113 4; 114 3;

115 4; 116 7; 117 57; 118 6; 119 5;

120 1; 123 1; 124 1; 125 1; 126 6;

127 9; 128 3; 129 34; 130 6; 131 14;

132 3; 133 34; 134 5; 135 4; 136 1;

138 5; 139 1; 140 2; 141 3; 142 17;

143 34; 144 7; 145 7; 146 2; 147 208;

148 35; 149 23; 150 3; 151 2; 152 1;

153 1; 154 1; 155 1; 156 2; 157 6;

158 11; 159 5; 160 3; 161 10; 162 2;

163 6; 164 1; 165 1; 166 1; 167 1;

168 5; 169 36; 170 7; 171 5; 172 2;

173 45; 174 72; 175 20; 176 5; 177 3;

178 1; 180 1; 181 1; 182 1; 183 1;

184 2; 185 4; 186 3; 187 3; 188 1;

189 18; 190 6; 191 16; 192 3; 193 2;

196 1; 197 1; 198 4; 199 2; 200 2;

201 2; 202 2; 203 3; 204 49; 205 113;

206 25; 207 13; 208 2; 209 1; 213 1;

214 2; 215 4; 216 3; 217 175; 218 40;

219 19; 220 4; 221 7; 222 2; 223 1;

227 1; 228 1; 229 4; 230 3; 231 5;

232 2; 233 17; 234 6; 235 3; 236 1;

240 1; 241 2; 242 2; 243 7; 244 6;

245 7; 246 15; 247 6; 248 2; 249 1;

255 1; 256 3; 257 18; 258 6; 259 9;

260 4; 261 5; 262 2; 263 2; 264 1;

265 1; 271 1; 272 1; 273 1; 274 9;

275 4; 276 2; 277 8; 278 4; 279 3;

280 1; 285 6; 286 2; 287 2; 288 10;

289 6; 290 2; 291 6; 292 10; 293 3;

294 2; 301 1; 303 1; 304 1; 305 17;

306 7; 307 45; 308 13; 309 7; 310 1;

316 1; 317 2; 318 2; 319 4; 320 2;

321 2; 322 1; 324 2; 330 1; 331 7;

332 4; 333 25; 334 9; 335 6; 336 2;

337 1; 345 2; 346 2; 347 3; 348 2;

349 3; 350 1; 351 1; 358 1; 359 3;

360 1; 361 1; 362 1; 374 3; 375 55;

376 19; 377 11; 378 18; 379 6; 380 3;

381 1; 390 1; 391 4; 392 2; 393 1;

394 1; 395 1; 406 1; 407 1; 419 1;

421 1; 422 1; 423 2; 424 1; 435 2;

436 1; 437 1; 448 1; 449 3; 450 1;

451 1; 452 1; 465 1; 466 1; 467 2;

468 1; 469 1; 539 1; 593 1; 594 2;

595 2; 596 1; 597 1;
